# Construction of feasible and accurate kinetic models of metabolism: A Bayesian approach

**DOI:** 10.1038/srep29635

**Published:** 2016-07-15

**Authors:** Pedro A. Saa, Lars K. Nielsen

**Affiliations:** 1 Australian Institute for Bioengineering and Nanotechnology (AIBN), The University of Queensland, Brisbane, QLD 4072, Australia

## Abstract

Kinetic models are essential to quantitatively understand and predict the behaviour of metabolic networks. Detailed and thermodynamically feasible kinetic models of metabolism are inherently difficult to formulate and fit. They have a large number of heterogeneous parameters, are non-linear and have complex interactions. Many powerful fitting strategies are ruled out by the intractability of the likelihood function. Here, we have developed a computational framework capable of fitting feasible and accurate kinetic models using Approximate Bayesian Computation. This framework readily supports advanced modelling features such as model selection and model-based experimental design. We illustrate this approach on the tightly-regulated mammalian methionine cycle. Sampling from the posterior distribution, the proposed framework generated thermodynamically feasible parameter samples that converged on the true values, and displayed remarkable prediction accuracy in several validation tests. Furthermore, *a posteriori* analysis of the parameter distributions enabled appraisal of the systems properties of the network (e.g., control structure) and key metabolic regulations. Finally, the framework was used to predict missing allosteric interactions.

Mathematical models are essential to unravel the complexity of metabolic networks. Within the vast range of models capable of describing different metabolic processes, kinetic models are the most suitable for predicting the behaviour of enzymatic reactions upon genetic and environmental perturbations^1^. Despite their obvious virtues, fitting of detailed kinetic models is challenging. Although there has been progress in the construction of mechanistic kinetic models describing particular metabolic pathways such as glycolysis in yeast^2^,^3^ and the central carbon metabolism of red blood cells^4^,^5^ and *E. coli*^6^,^7^, all efforts suffer from common problems and limitations, namely^8^: 1) difficult identification of highly-parameterized models, 2) use of *in vitro* kinetic data to fit and approximate enzyme kinetics *in vivo*, and 3) inherently complex nonlinear nature of mechanistic rate laws. Ideally, one would like to globally fit detailed mechanistic models of metabolic pathways using *in vivo* data directly (e.g., following time-course metabolite data^9^), however strong parameter coupling and homeostatic control render this task impossible with the exception of small observable pathways (e.g., glycolysis^10^). The use of simplified kinetics has been explored extensively to circumvent this limitation. Depending on the scope, application and particular model features, formalisms such Generalized Mass Action^11^, Log-Lin kinetics^12^, among others^13^,^14^,^15^, have been employed to study the dynamic behaviour of metabolic networks (for a detailed description refer to^8^). Although integration of these formalisms with various kinetic modelling frameworks has yielded valuable insights into the operation of metabolic networks^16^,^17^,^18^, they offer limited predictive power due to their lack of kinetic detail. Complex kinetic features such as allosteric regulation are recognized as fundamental to explain complex metabolic behaviours *in vivo* such as rapid microbial metabolic adaptations^19^,^20^. As such, the challenge of constructing detailed kinetic models from *in vivo* data remains a central problem in the field^1^,^8^.

Detailed and thermodynamically feasible kinetic models of metabolism have a large number of heterogeneous parameters, are non-linear and have complex relations. Many powerful fitting strategies are ruled out by the intractability of the likelihood function. However, Approximate Bayesian Computation (ABC) can overcome this limitation by sampling from an approximation of the posterior distribution without explicitly evaluating the likelihood function^21^. In this study, we use an ABC-based sampling framework that formalizes and substantially extends the heuristic ‘Ensemble Modelling’ (EM) approach for kinetic model construction and parameter fitting^22^,^23^,^24^,^25^,^26^. While EM is more similar to a stochastic optimization approach aimed at finding one^22^,^24^ or several^23^,^25^,^27^ accurate parameter sets, our approach is based on Bayesian statistics and seeks to determine the joint parameter distribution (i.e., posterior distribution) capable of explaining the data. We have greatly expanded the range of kinetics covered from non-allosteric compulsory-order kinetics in EM, to both general non-allosteric – i.e., sequential and random-order–and allosteric kinetics using our General Reaction Assembly and Sampling Platform (GRASP)^28^. We note the latter platform constitutes a convenient prior for Bayesian inference in kinetic modelling, as it incorporates prior knowledge in the form of thermodynamic relationships, thereby ensuring the feasibility of the sampled kinetic parameters. To the best of our knowledge, this work constitutes the first attempt to construct feasible and detailed kinetic models of metabolism using Bayesian inference. Importantly, it provides a statistically sound framework for parameter inference, prediction intervals and model selection. Using the most experimentally supported model of the tightly-regulated mammalian methionine cycle as a surrogate biological system^29^, we demonstrate that the proposed framework: 1) converges to the ‘true’ parameter values, 2) accurately predicts metabolic states upon genetic perturbations, 3) reveals emergent properties of the system, and 4) suggests missing metabolic interactions. This work illustrates the core capabilities of this framework for the construction of detailed kinetic models, however it holds a number of possible further applications. In particular, experimental design and integration of information from enzyme databases or previous kinetic reconstructions into the prior, emerge as important applications that can be properly addressed using this Bayesian approach.

## Results

### ABC for constructing feasible and accurate kinetic models of metabolism

Bayesian inference derives the *a posteriori* or posterior distribution *p*(**θ***|***y**) from an *a priori* or prior probability distribution *p*(**θ**) for the parameters **θ** by updating with experimental observations **y** through the likelihood *p*(**y**|**θ**) using Bayes rule^30^


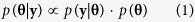


**Figure 1:**
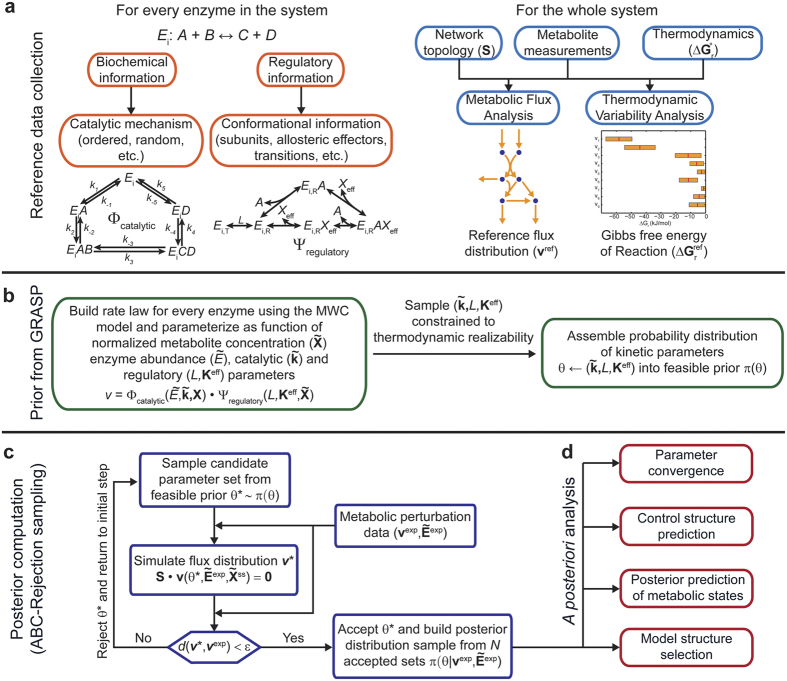
General workflow for building feasible and detailed kinetic models employing a Bayesian approach. (**a**) Collection of available biochemical, thermodynamic and structural data for all the components of the system. Reference metabolite concentrations ranges and metabolic fluxes are also needed to define the reference point. (**b**) Generation of a feasible prior distribution of kinetic parameters (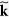,*L*,**K**^eff^) using GRASP. This distribution is implicitly described by the auxiliary parameters 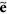 (enzyme intermediate abundances), **R** (microscopic reversibilities) and **r**_elem_ (branching vector), and spans the full kinetic space allowable by the reaction mechanism, thermodynamics, structural and flux data at the reference state. (**c**) Computation of the posterior parameter sample is achieved by employing a rejection sampling scheme based on Approximate Bayesian Computation. (**d**) *A posteriori* analysis of the posterior distribution enables assessment of parameter convergence, control structure prediction, prediction of metabolic states upon genetic and environmental perturbations, and exploration of possibly missing metabolic interactions.

Detailed kinetic models have non-universal rate laws with complex parameter support and there is no simple parameterization of the likelihood that is computationally tractable. Approximate Bayesian Computation is a class of likelihood-free computational methods for Bayesian Inference. ABC requires an efficient strategy for sampling the prior distribution and we used our recently published framework GRASP to sample the range of thermodynamically allowable enzymatic behaviours (Fig. 1a,b). Briefly, GRASP relies on the mechanistic Monod-Wyman-Changeux (MWC) framework^31^ to generate feasible kinetic parameterizations consistent with biochemical, structural and thermodynamic reference data. Exhaustive exploration of the feasible parameter space is achieved by re-parameterizing the kinetic parameters (rate constants 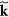, allosteric constant *L*, effector constants **K**^eff^), as function of readily sampled auxiliary variables (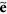 enzyme intermediate abundances, **R** microscopic reversibilities and **r**_elem_ branching vector). The reader is referred to Methods for further details. ABC methods avoid likelihood evaluation by proposing parameters from the prior, simulating data from the model conditional on those parameters, and accepting those parameters that simulate data close to the observed values (Fig. 1c, refer to Methods for details). As a proof-of-principle, the simplest ABC-based sampler – the rejection sampler^32^–was implemented. Once enough parameter samples have been accepted, the posterior distribution is assembled. A wide array of *a posteriori* analyses can be performed employing this distribution (Fig. 1d). The application of this approach is next illustrated in the study of the mammalian methionine cycle.

### Assessing convergence of sampled parameters: the methionine cycle as a case study

**Figure 2:**
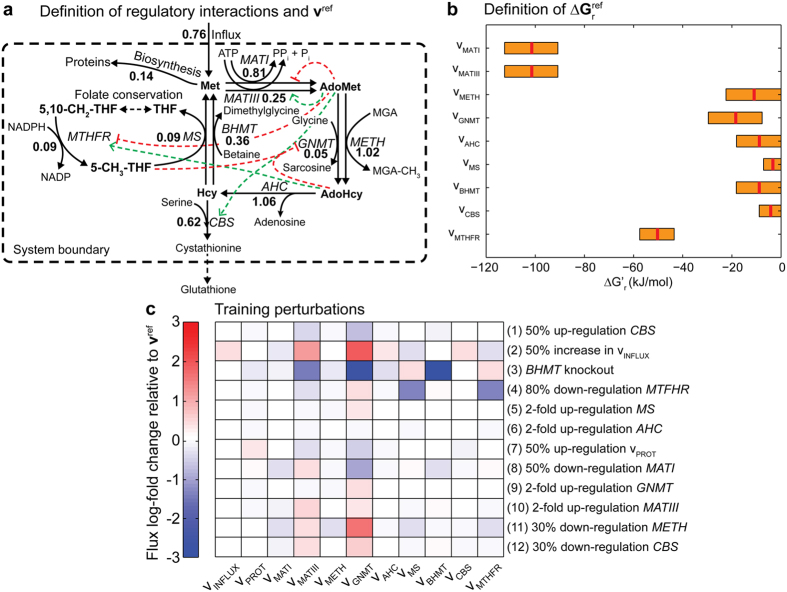
Modelling the mammalian methionine cycle. (**a**) Schematic representation of the methionine cycle and its main regulatory interactions. The modelled metabolites are shown in bold, whereas the red and green dashed lines represent known inhibitory (red) and excitatory (green) interactions within this cycle. The enzyme names are displayed in italics. Abbreviations: Met, methionine; AdoMet, S-adenosylmethionine; AdoHcy, S-adenosylhomocysteine; Hcy, homocysteine; 5-CH_3_-THF, 5-methyltetrahydrofolate; 5,10-CH_2_-THF, 5,10-methylenetetrahydrofolate; THF, tetrahydrofolate; MGA, methyl group acceptor; MGA-CH_3_ methyl bound to the methyl group acceptor; MATI, methionine adenosyltransferase I; MATIII, methionine adenosyltransferase III; GNMT, glycine-N-methyltransferase; METH, S-adenosylmethionine-dependent methyltransferase; AHC, adenosylhomocysteinase; BHMT, betaine homocysteine S-methyltransferase; MS, methionine synthase; MTHFR, methylenetetrahydrofolate reductase; CBS, cystathionine β-synthase. Bold numbers denote the reference flux distribution of this cycle as calculated by the true model (see Methods). (**b**) Estimated Gibbs free energy of reactions ranges for each reaction in the cycle. The red lines represent the mean Gibbs free energy of reactions which were used to define the reference thermodynamic point. (**c**) Heat map of the log-fold change in the flux responses of 12 simulated perturbations relative to the reference flux distribution (**a**). These experiments represent one-at-a-time genetic and/or environmental perturbations in the reaction fluxes of this cycle. The details of these perturbations are shown in Supplementary Table S1.

The methionine cycle links essential trans-methylation and trans-sulphuration reactions responsible of maintaining cellular methylation and antioxidant homeostasis^33^. Proper functioning of this cycle is essential for growth and development, and its abnormal operation is associated with complex diseases, such as cardiovascular^34^ and liver diseases, neural tube defects^35^ and cancer^33^,^36^,^37^. Due to its central role in normal growth and metabolism, several mathematical models have been developed describing some of the particular regulatory features underpinning its operation^29^,^38^,^39^,^40^,^41^,^42^. A realistic representation of the mammalian methionine cycle thus constitutes a suitable benchmark to exemplify our approach (Fig. 2a). Previous studies have shown that metabolic perturbations in some of its constituents generate non-intuitive complex responses, such as switching between parallel reactions upon methionine intake variations^29^, and bistability in S-adenosylmethionine as a consequence of the metabolic regulation of *MATI* and *MATIII* and the cooperative kinetics of *GNMT*^38^.

Our workflow starts by assembling the reference data necessary to sample the kinetic parameters of this cycle, namely biochemical data, structural information, reference flux distribution (**v**^ref^) and Gibbs free energies of reaction 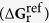 (Fig. 2a,b and Supplementary Table S2). Knowledge of the latter quantities is essential as the parameterization is built around this operation point. Although the exact value of 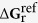 typically cannot be exactly determined, feasible ranges can be estimated (see Methods). Here, the mean Gibbs free energy of each reaction was considered as a representative measure of the thermodynamic reference state of the system, although it is equally possible to sample this quantity. The same consideration applies for **v**^ref^. Even when it is not possible to exactly determine its values, a representative flux distribution can always be defined based on exchange rates and 13C-labelling data^24^. Preliminary thermodynamic examination of the cycle showed that 4 out of the 9 reactions (*v*_MATI_, *v*_MATIII_, *v*_GNMT_ and *v*_MTHFR_) are strictly irreversible at the reference point, and as expected, the highest thermodynamic driving force is displayed by the initial steps of the cycle (*v*_MATI_ and *v*_MATIII_). Regarding the reversible reactions, only *v*_CBS_ and *v*_MS_ could potentially change their direction as they operate close to equilibrium and the network topology allows it. However, the high thermodynamic driven force of the first steps of this pathway means that this cycle in practice operates in the canonical direction depicted in Fig. 2a.

Once the reference information has been gathered, computation of the posterior is achieved by adding experimental information into the sampling. To evaluate the performance and convergence of our approach, 12 metabolic perturbations were simulated (Fig. 2c, details in Supplementary Table S1) using a reference model based on the most detailed and experimentally supported description of this cycle (see Methods). Henceforth, this model will be considered the ‘true model’. It is important to highlight though that this model was built using approximate formulae mostly fitted to *in vitro* data (Supplementary Text S1), and hence, differences between our detailed parameterization and the ‘true model’ responses are to be expected. Notably, the proposed parameterization contains 72 parameters (29 more parameters than the true model) describing the kinetic behaviour of this pathway. The main differences between the two descriptions relate to the application of common assumptions and simplifications in the parameterization, namely: reaction irreversibility (*v*_PROT_, *v*_MATI_, *v*_METH_, *v*_BHMT_ and *v*_MS_), i.e., no backward catalysis (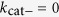), fast equilibrium kinetics (*v*_AHC_), and simplified formulae for enzymes undergoing allosteric regulation (*v*_MATIII_, *v*_GNMT_, *v*_CBS_ and *v*_MTHFR_).

**Figure 3:**
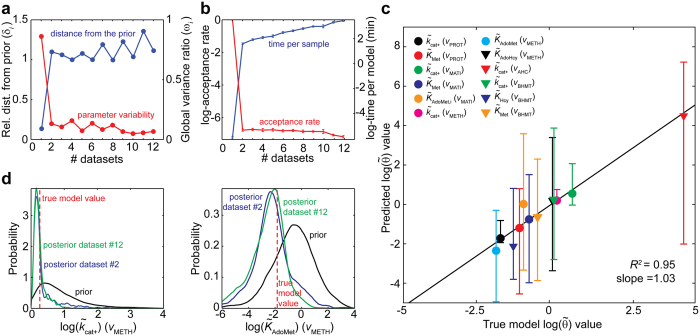
Assessment of parameter convergence in the methionine cycle. (**a**) Convergence of main posterior statistics as a function of dataset size. The blue line represents the Euclidian distance between the prior and the posterior parameter expectancy for different experimental sets. As the dataset increases, the relative distance between the expectancies increases. On the other hand, the red line represents the Euclidean distance between the posterior and prior standard deviation ratio. As more information is known, the overall variability decreases. (**b**) Sampling performance of the ABC algorithm for different datasets sizes. The red and blue lines denote the average acceptance rate and time per accepted parameter set as data increases, respectively. In each simulation, 10^3^ parameter sets were generated in parallel using 10 computing cores. The error bars represent two standard deviations for each *in silico* experiment. (**c**) Comparison of the true and expected parameter values for all comparable kinetic parameters in the methionine model. Each symbol represents the expected parameter value, whereas the error bars describe the 95%-credible intervals for the respective parameter. There is high agreement between the predicted parameter values and the parameters from the true model (*R*^2^ = 0.95, slope = 1.03). (**d**) Illustration of the progression of the marginal posterior distribution of the *METH* kinetic parameters (*k*_cat+_ and *K*_AdoMet_) for different experimental sets. In both cases, the distributions converge rapidly to the true parameter value as more data becomes available.

A good estimator should converge on the true parameter as more experimental data are included. Convergence was here tested by including the 12 simulated experimental datasets one-at-a-time. In complex non-linear systems, the information contribution by individual experiments varies considerably. In the current simulated experimental series, the second perturbation included (50% increase in *v*_INFLUX_) contributes by far the most information (Fig. 3). This result was also observed when adding the first three datasets in different orders (Supplementary Fig. S1). Introducing the second dataset shifted the solution significantly from the prior and dramatically reduced parameter variability (Fig. 3a). The high information content in the *v*_INFLUX_ experiment is consistent with a previous study demonstrating that fluctuations in the methionine influx result in non-trivial switching between parallel reactions leading to substantial flux rearrangements in this cycle^29^. Informative perturbations are those where only a minor portion of prior samples are consistent with the observed behaviour. Computationally, this was reflected in a steep drop in the acceptance rate, and consequently, a significant rise in the sampling time per model (Fig. 3b). Subsequent additions of datasets change these parameters far less dramatically.

The fitted parameters were compared with parameters in the true model. Only the canonical parameters (i.e., *K*_S_ and *k*_cat_) from reactions *v*_PROT_, *v*_MATI_, *v*_METH_, *v*_BHMT_, *v*_MS_ and *v*_AHC_ (12 in total) could be contrasted, as the parameterization differs greatly for the remaining allosteric reactions (Fig. 3c). The expected value of the final estimates were close to the true value and always within the 95%-credible interval. Progression of the posterior after two and twelve datasets is illustrated here for the *k*_cat+_ and *K*_AdoMet_ parameters of *v*_METH_ (Fig. 3d), while full progressions of the marginal distributions of each parameter is shown elsewhere (Supplementary Fig. S2). Notably, the most uncertain parameter is *k*_cat+_ of the AHC reaction, which was originally assumed to reach fast equilibrium (Supplementary Text S1). As the latter parameterization disagrees with our parametric form, higher uncertainty is expected for this particular parameter. More importantly, inspection of the posterior distribution enables detection of such situations which could be addressed in subsequent refinement experiments.

### Posterior samples accurately capture the underlying control structure of the network

We have demonstrated convergence of the approach. However, the credible intervals for the parameters remain large and it is important to evaluate the predictive powers given this level of uncertainty. In the following, we employ the posterior distribution to predict the control structure of the methionine cycle. The control exerted by each enzyme on every reaction of the network can be quantified through flux control coefficients derived from Metabolic Control Analysis (MCA)^43^,^44^.

**Figure 4:**
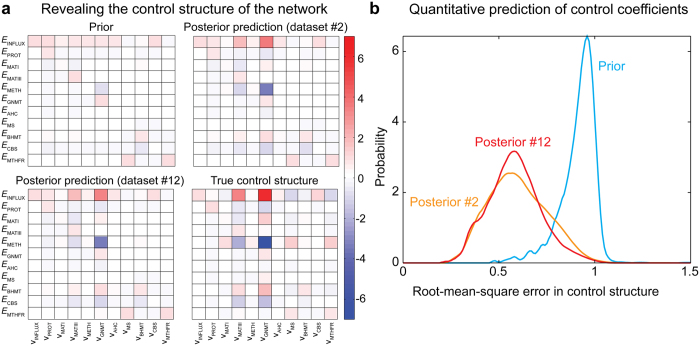
Revealing the control structure of the methionine cycle. (**a**) Comparison of the true and predicted control structure of the methionine cycle at the reference state employing the prior and the posteriors derived from dataset #2 and #12 (full dataset). The heat maps represent the flux control coefficient magnitudes for each enzyme in the network. The progressive addition of data improves prediction of the dynamic behaviour of the network encoded in the control structure. (**b**) Quantitative assessment of the flux control coefficient predictions for the prior and the above posteriors. This plot depicts the root-mean-square error distributions for the prior and posterior predictions of the flux control coefficients magnitudes. Addition of new experimental data substantially increases the accuracy of the posterior predictions compared to the prior prediction.

The control structures estimated from the prior, posterior with 2 observations (dataset #2) and posterior with 12 observations (dataset #12) were computed and compared with the true control structure of the network (Fig. 4a). Although the prior captures elements of the control structure, predictions improve significantly as more data is included. Qualitatively, the prior by itself exhibits surprisingly good predictions of the signs of flux control coefficients (Matthew’s correlation coefficient of 0.638; Supplementary Fig. S3). Quantitatively, however, the expected root-mean-squared error dropped from 0.94 in the prior prediction to 0.59 and 0.58 for the posterior #2 and #12, respectively (Fig. 4b). We have further assessed the stability of the posterior distribution by checking the stability of the Jacobian matrix under three different conditions, namely: the reference state condition, condition #2 and condition #12. The totality of the parameter sets yielded stable models under the different conditions tested (Supplementary Fig. S4). Overall, these results show that reasonably and increasingly accurate predictions of the underpinning control structure of metabolic networks can be attained without sacrificing complexity or compromising thermodynamic feasibility.

### Predicting metabolic states upon genetic and environmental perturbations

**Figure 5:**
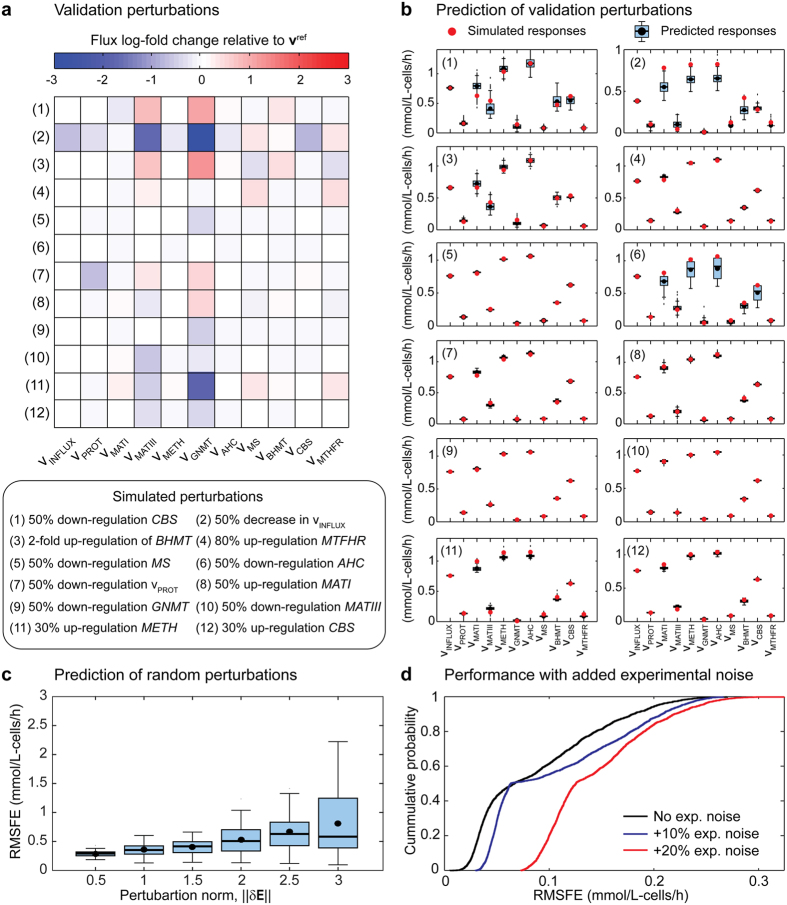
Posterior prediction of metabolic states. (**a**) Heat map of the log-fold change in the flux responses of 12 validation perturbations relative to the reference flux distribution. These perturbations are orthogonal to the perturbations shown in Fig. 2c and describe one-at-a-time genetic and/or environmental perturbations in the reactions of this cycle. Numerical values of each perturbation are found in Supplementary Table S5. (**b**) Posterior predictions for the 12 validation perturbations. Boxplots represent the posterior predictive distributions whereas the red circles describe the true model responses. There is overall good agreement between predicted and true model responses. (**c**) Posterior predictions for 50 random enzyme level perturbations of magnitudes (δ**E**). Boxplots represent the root-mean-square flux error (RMSFE) distribution for perturbations of different magnitudes. The trained model exhibits an overall robust predictive performance across the tested conditions. (**d**) Assessment of the impact of experimental noise on the predictive power of the posterior distribution. The probability distributions of the RMSFE simulating three conditions are shown and compared. The addition of experimental noise does not substantially affect the prediction of the sampled models.

The key feature of kinetic models is their capacity to predict the dynamic behaviour of metabolic networks as a function of protein (enzyme) and metabolite concentrations. In order to evaluate the predictive performance and robustness against experimental noise of the current approach, a validation dataset was generated comprising 12 metabolic perturbations of the same magnitude but in the opposite direction to the training set (Supplementary Table S5) (Fig. 5a). Notably, opposing perturbations of similar magnitude provoked significantly different outcomes owing to the inherently nonlinear nature of the system (compare Figs 2c and 5a). Figure 5b depicts the predictions obtained from the full training dataset for each perturbation. Slightly worse results are obtained if the training set #2 is chosen (Supplementary Fig. S5 and Table S6). There is overall very good agreement between our predictions and the true responses. The 50% down-regulations of *CBS* and *AHC* and the 50% decrease of *v*_INFLUX_ display relatively larger deviations from the true mean (0.070, 0.114 and 0.092 mmol/L-cells/h, respectively) than the remaining 9 cases (average deviation between 5·10^−3^–5·10^−2^ mmol/L-cells/h). Critically, large deviation is in all cases associated with broader credible intervals, i.e., the greater deviation can be inferred from the actual fit. Interestingly, the higher observed disagreement does not correlate with a higher response in the true system. While the 50% down-regulation of *CBS* exerts important flux changes relative to the reference state, the 50% down-regulation of *AHC* shows almost no apparent flux redistribution (Fig. 5a). Instead, the greater variability correlates with parameter uncertainty, i.e., more complex enzymes display more variable behaviours in the worst prediction cases.

The simulations illustrate the classical MCA observation that regulatory enzymes are not generally flux-controlling enzymes^44^, and thus their manipulation does not commonly yield much information^45^. Perturbation of regulatory enzymes (i.e., *v*_MATI/III_, *v*_GNMT_, *v*_CBS_ and *v*_MTHFR_) had limited effect on the flux responses. In contrast, flux controlling-reactions like *v*_INFLUX_ provides more interesting insights (refer to Fig. 4a). We note that inspection of the predictive posterior behaviour reveals useful information for experimental design. Perturbations displaying more variability–and hence higher uncertainty–yield the highest information gain about the system’s behaviour. In this particular case, manipulations targeting *v*_CBS_, *v*_INFLUX_ and/or *v*_AHC_ will be more informative than perturbations targeting the response of *v*_BHMT_, *v*_MATI/III_, *v*_PROT_, *v*_MS_, *v*_GNMT_, *v*_METH_ or *v*_MTHFR_.

We further tested the predictive power of our model by performing random perturbations in different directions (δ**E)** on the enzyme levels (Fig. 5c). Perturbation directions from the δ-norm hypersphere were randomly sampled using the Marsaglia method, i.e., generate independent Gaussian random perturbations for each reaction with zero mean and unit variance, and then normalizing the perturbation vector to a δ-length. Once the direction of the perturbation is generated, the enzyme level of reaction *i* is computed as 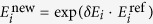, where 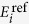 denotes the reference enzyme level (equal to 1) and *δE*_*i*_ denotes the enzymatic perturbation of the corresponding enzyme. Figure 5c shows the root-mean-square flux error (RMSFE) of our model for 50 random perturbations at different perturbation magnitudes. As expected, as the magnitude of the perturbation increases, the expected RMFSE increases. Notably, the median of the RMSFE remains rather stable around 0.5 mmol/L-cells/h, suggesting a robust predictive performance of our model even for large perturbations.

Finally, we assessed the robustness of our approach against experimental noise. To this end, new reference and experimental fluxes were simulated with 10% and 20% deviation from the true values (Supplementary Table S7) and the sampling step was repeated using dataset #2 as training set. The addition of experimental noise did not catastrophically impact the predictive power of the posterior distribution (Fig. 5d). For example, the RMSFE 95%-percentile increased from 0.203 to 0.226 mmol/L-cells/h (~11.3% deviation from the base case) when 10% experimental noise was considered, and to 0.244 mmol/L-cells/h (~20.2% deviation from the base case) when 20% was included. This proportional increase is however variable in the error distributions, displaying higher discrepancies at lower percentiles, i.e., lower confidence (Supplementary Table S8). More importantly, at higher confidence, e.g., 99%-percentile, the deviations change to approx. 5.5% and 14.8% for the 10% and 20% noise additions, respectively.

### Unveiling missing regulatory interactions

Robust metabolic control is mainly achieved through allosteric regulation^46^. The activity of allosteric enzymes can be greatly modified by the binding of an effector to an allosteric site different from the active site. These interactions are essential for metabolic adaption upon sudden environmental changes^20^ and for maintaining metabolic homeostasis^46^. The methionine cycle represents a good example of this type of metabolic control. The activity of the regulatory enzymes *MATIII* and *GNMT* varies greatly upon perturbations on the methionine uptake (Fig. 2c).

**Figure 6:**
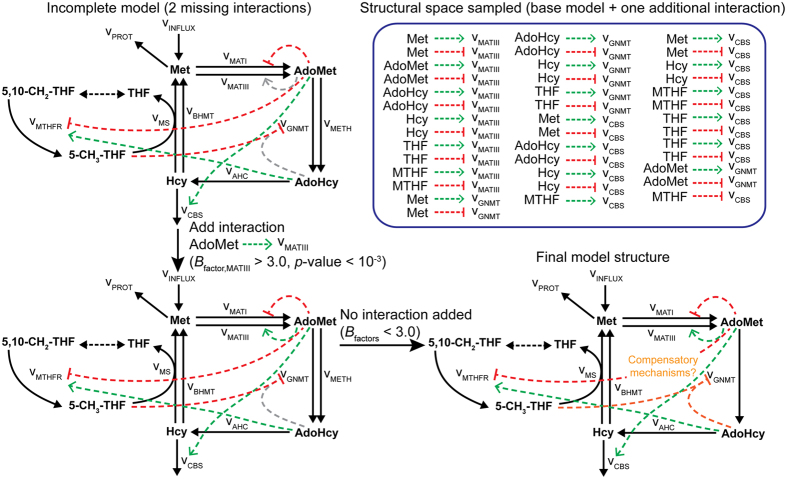
Unveiling possibly mossing regulatory interactions. Exploration of likely missing allosteric interactions in the methionine cycle. Positive and negative interactions are represented by green and red arrows respectively, whereas missing interactions are represent by grey arrows. Two sequential additions were performed but only the positive activation of *MATIII* by AdoMet was recovered from the incomplete model (*p*-value = 3.2·10^−4^ < 10^−3^). Addition of the negative inhibition of *GNMT* by AdoHcy was not found to be a significant interaction despite its presence in the original model (Bayes factors <3.0). This could point to the presence of compensatory mechanisms in the network (see Supplementary Fig. S6).

While reactions of enzymes are known, the identification of particular allosteric sites from protein structure remains a great challenge^47^,^48^. An alternative strategy is to use metabolic network models to unravel possibly missing allosteric interactions. Given a possibly incomplete model, the objective is to determine which interactions may be missing. Implementation of this task is straightforward within the Bayesian setting and it is commonly known as (Bayesian) model selection (see Methods). Briefly, a set of possible model structures are defined describing different allosteric interactions and each structure is given equal probability of being selected by the ABC algorithm. The frequency of each structure in the posterior is proportional to its likelihood to be chosen. To illustrate an application of this framework, we deleted the feedback activation of *v*_MATIII_ by AdoMet and the feedback inhibition of *v*_GNMT_ by AdoHyc from the original model (Fig. 6). In this case, the full dataset (dataset #12, Fig. 2c) was employed to perform model selection given the increased size of the parameter space (41 additional parameters describing all possible structures).

Starting from the incomplete model structure as the reference structure, we explored the most likely interaction to be added. The model selection algorithm yielded the positive activation of *MATIII* by AdoMet as the most likely interaction to be included in the model (Bayes factor >3, *p*-value = 3.2·10^−4^; Supplementary Table S9). After including this interaction, the above procedure was repeated but now using the augmented structure as reference. No further interaction was significantly enriched in the posterior (Bayes factors <3; Supplementary Table S10). Specifically, the mild inhibition of *v*_GNMT_ by AdoHyc incorporated in the true model based on experimental data^49^ was missed.

Interactions may be missed due to redundancy, i.e., the existence of alternative mechanisms to achieve similar regulation. Regulation of *GNMT* is essential, particularly under high methionine concentrations^29^ (Supplementary Text S1). In addition to AdoHyc inhibition, *GNMT* is also under non-competitive inhibition by 5-CH_3_-THF^50^ (Fig. 6). Moreover, the enzyme is under strong positive homotropic regulation (positive cooperativity) by the substrate, AdoMet (*n*_Hill_ = 2.3)^51^. With the current simulated experiments, there was no preference for models containing either 5-CH_3_-THF or AdoHyc inhibition over a model containing neither (i.e., relying on homotropic regulation only) (Supplementary Fig. S6 and Table S11). In this particular case, a more straightforward approach for unravelling the correct model structure would be to individually test the candidate interactions *in vitro*. The present framework then offers a rational method for proposing testable hypothesis about the regulation of metabolic networks.

## Discussion

Kinetic models convey detailed information about the mechanisms underpinning the modulation of enzymatic fluxes to the cell needs. Continuous advances in omics technologies have enabled the collection of high-quality datasets for building these models. However, the construction and fitting of detailed kinetic models remains a major ongoing challenge in the field^1^,^8^.

In this study, we successfully cast the fitting problem as an ABC-sampling problem using the GRASP as starting prior to construct detailed, feasible kinetic models for each enzyme (Fig. 1). We exemplified the application of this approach to the mammalian methionine cycle (Fig. 2), demonstrating its capabilities for fitting predictive models. Detailed kinetic models are difficult to fit as they contain many, highly correlated parameters. For example, our detailed parameterization required 72 parameters to mechanistically describe the methionine cycle, as opposed to the 43 parameters from the original model. Despite the high number of parameters, analysis of the posterior distribution demonstrated that expected parameter values converged to the true values, albeit their marginal distributions remained fairly broad (Fig. 3c,d). It is well recognized that parameters from quantitative non-linear models are difficult to fit collectively from *in vivo* data, often yielding large parameter uncertainties. Indeed, a previous model survey showed that most biological models display a “sloppy” spectrum of parameter sensitivities^52^. However, as noted by the above authors, even when parameters are not tightly fitted, tight quantitative predictions can be obtained and later verified experimentally. For instance, a globally fitted growth-factor-signalling network model of PC12 cells yielded fairly accurate and testable predictions of the system’s behaviour using a modest training set^53^. This is also the case of our parameterization for predicting the behaviour of the system upon new perturbations (Fig. 5a,b) as well as for revealing the control structure of the network (Fig. 4). Furthermore, simulation of experimental noise did not substantially deteriorate the predictive power of our strategy (Fig. 5c). These results demonstrate the robustness of our approach and also encourage the use of detailed kinetic parameterizations grounded on thermodynamic and kinetic fundamentals. Using global sampling of thermodynamically feasible parameter sets, valuable insights about the properties of the system can be gained even in the absence of data (Fig. 4a). These insights would be most likely lost if using simplified parameterizations to ease global fitting, as they miss important thermodynamic relationships and are often only locally valid. Rather than focusing on the precise identification of parameters from simplified “sloppy” models–which will commonly remain poorly constrained even when extensive data is available^54^–more attention should be placed on the actual model predictions. For the construction of such predictive models, our approach offers an attractive alternative.

ABC lends itself to more advanced modelling tasks such as model selection and experimental design^55^. Unlike frequentist hypothesis testing, Bayes factors enable: 1) comparison of models with entirely different structures rather than only nested models, and 2) weighting the evidence against and in favour of a hypothetical model^56^. Application of this measure for topological discovery of metabolic signals has lately yielded valuable insights into the regulation of different signalling pathways^57^,^58^,^59^. Here, we have demonstrated the use of this simple measure for testing the inclusion (or exclusion) of speculative allosteric interactions (Fig. 6). In the context of experimental design, our results showed that a single experiment (dataset #2) contributed the majority of information to the fit (Fig. 3a,b). This highlights the challenge of designing informative experiments for networks with highly non-linear kinetics and often unknown interactions. We note that our approach can be readily adapted for rational experimental design. For instance, analysis of posterior predictions suggests that targeting reactions *v*_AHC_, *v*_INFLUX_, or *v*_CBS_ in future experiments will be more informative of the behaviour of system, as they display relative larger uncertainty in their responses (Figs 5b and 6). Another useful capability of this Bayesian framework is the possibility to refine the prior with partial information from enzyme databases or previous kinetic reconstruction efforts. We have recently reported an efficient approach for including known enzymatic data (i.e., *k*_cat_ and/or *K*_S_) within GRASP, greatly improving kinetic descriptions of single-enzymes under different conditions^60^. Refinement of the prior with kinetic information may prove particularly beneficial for improving parameter identification in larger models.

Scale is the major challenge and potential limitation of sampling-based strategies. There are more efficient ABC implementations displaying accelerated convergence^61^,^62^,^63^. However, application of such algorithms is not exempt of difficulties, particularly in multi-dimensional models. Posterior estimates inevitably deteriorate with increasing dimensionality^64^. Implementation of ‘divide and conquer’-type strategies, i.e., breaking the model into smaller models or modules, could be an effective strategy to fit larger models^65^. Considering the universally sloppy sensitivities of most biological models^52^, application of such strategies could not be only suitable, but critical for the development of large-scale kinetic models.

## Materials and Methods

### Methionine cycle model

This model describes the mass-balances for the main intracellular intermediates involved in the cycle, namely: methionine (Met), S-adenosylmethionine (AdoMet), S-adenosylhomocysteine (AdoHcy), homocysteine (Hcy), methyl-tetrahydrofolate (MTHF) and 5,10-methylenetetrahydrofolate (THF), and it also includes a moiety conservation equation (folate pool conservation). The model is primarily based on the most detailed description^29^. While most of the original structure was conserved, two changes were introduced to further improve its consistency with recent experimental data: 1) the adenosyl-homocysteinase reaction (*v*_AHC_) was not considered at equilibrium, but instead the rapid equilibrium expression proposed by Reed *et al*.^40^ was used, and 2) the methionine uptake flux (*v*_INFLUX_) was set to 0.76 mmol/L-cells/h in agreement with recent experimental findings^66^. A detailed description of the model is provided in Supplementary Text S1. The reference model of the methionine cycle has been deposited in the BioModels database^67^ identifier MODEL1603150000.

### Assembly of a feasible prior using GRASP

We have previously developed a systematic framework (GRASP)^28^ capable of parameterizing and sampling complex kinetics consistent with thermodynamics and using minimal reference data. The employed parameterization rests on the fundamentals of the Monod-Wyman-Changeux model^31^, which enables description of the majority of kinetic behaviors found in oligomeric enzymes (both allosteric and non-allosteric). Formally, GRASP relies on the decomposition of the velocity of reaction as the product of two independent functions^68^,


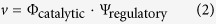


where 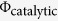 represents the rate law function for the enzyme subunits in the relaxed state (which only depends on the reaction mechanism), and 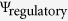 denotes a regulatory function describing the conformational transitions from the tense (T) to the relaxed state (R). Equation 2 recast the problem of parameterizing the kinetics of an enzyme into finding suitable parameterizations for the catalytic mechanism and – independently – the mechanism of allosteric transitions.

Firstly, the rate of reaction describing the catalytic mechanism of an enzyme can be derived using the Quasi-Steady-State Assumption (QSSA)^69^ for the enzyme intermediates. The resulting rate law describes the velocity rate of the enzyme as a function of the total enzyme concentration (*E*_total_), reactant concentrations (**X**) and rate constants (**k**). The latter will be ultimately responsible for the kinetic features of the enzyme. The space of allowable kinetics is then explored by GRASP using a sampling strategy that is consistent with thermodynamic constraints by employing a convenient normalization at the elementary reaction level^22^. This procedure scales the rate constants (denoted by 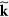) with respect to a reference point characterized by a reaction flux (*v*^ref^), thermodynamic driving force 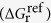 and enzyme concentration 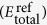. This normalization enables re-parameterization of 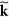 as a function of auxiliary parameters 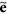 (enzyme intermediate abundances), **R** (microscopic reversibilities) and **r**_elem_ (branching vector), all three of which are bounded and hence readily sampled. A similar strategy is adopted to describe the mechanism of allosteric transitions. Within the MWC model, allosteric transitions are primarily described by the allosteric (*L*) and effectors dissociation (**K**^eff^) constants. These parameters can be calculated once the enzyme state abundances for the R and T states have been sampled^28^. As such, the distribution of (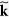, *L*, **K**^eff^) generated by uniformly sampling the auxiliary parameters (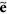, **R**, **r**_elem_) for each enzyme spans the full kinetic space consistent with the reaction mechanism, thermodynamics, measured rate and structural information. Importantly, this distribution constitutes a feasible prior to perform Bayesian Inference.

### Approximate Bayesian Computation: Rejection sampler

Bayesian inference employs Bayes theorem (Equation 1) to update the posterior distribution *p*(**θ**|**y**) using the prior distribution *p*(**θ**) and likelihood function *p*(**y**|**θ**). Despite the simplicity of this expression, it is rarely possible to compute posterior without the help of Monte Carlo simulation. In fact, formulation of the likelihood function might not be even possible considering the potentially high coupling between kinetic parameters. ABC copes with this limitation by “rejection sampling”, i.e., sampling parameter sets (particles), simulate outcomes given these parameters sets, and keeping only those parameter sets that simulate data within a certain tolerance *ε*_T_^21^. The discrepancy between the model and the experimental data can be measured using suitable distance functions, e.g., Euclidean distance, taxicab norm, infinite norm, etc. We have chosen a weighted infinite-norm distance to compare the simulated (**v**^sim^) and experimental (**v**^exp^) fluxes (Equation 3). The weighting term captures the appreciable differences in the order of magnitude between fluxes (up to approx. 22-fold for **v**^ref^), ensuring reasonable fit for all reactions. The overall tolerance *ε*_T_ was set to 0.2 showing a good compromise between accuracy and convergence time. Finally, we ran the rejection algorithm until *N* = 10^3^ particles were accepted. Preliminary results using higher sample sizes (e.g., 2 · 10^3^ and 5 · 10^3^) showed no significant changes in the summary statistics of the kinetic parameters (Supplementary Fig. S7), and 10^3^ particles were deemed adequate for parameter inference.


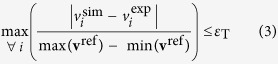


### Predictive posterior expectancy

The posterior distribution can be used to predict the likely state of the system upon perturbations taking into account the parameter uncertainty. Particularly, we are interested in predicting the expected value of a function Π, which might depend on both the model parameters **θ** and data **y**. The latter expectation can be calculated as follows^30^,


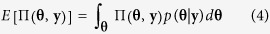


For the special case of predicting the outcome of genetic perturbations, we want to predict the expected outcome of perturbations y^*^ given the data at hand, i.e., *p*(y^*^|**y**). The latter is achieved by replacing Π(**θ**, **y**) for *p*(y^*^|**y**) in Eq. (4) and sampling from the resulting expression. Monte Carlo simulation can be employed to numerically sample the latter. Briefly, θ^*^ can be sampled from *p*(**θ**|**y**) and then used to simulate y^*^ from **S** · v^*^(y^*^) = **0**. The pairs (θ^*^|y^*^) are drawn from the joint distribution *p*(**θ**, y^*^|**y**), and thus y^*^ is a draw from *p*(y^*^|**y**). Similarly, other useful statistics and quantities (e.g., control coefficients) can be computed following this procedure.

### Parameter convergence

Assessment of parameter convergence was achieved by computing the relative distance (Euclidian measure) between the posterior expected parameter values for each training set *i* 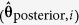 with the corresponding prior expectancy 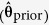, i.e., 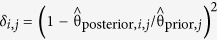, 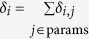. Expected parameter values were computed using Eq. (4) by setting 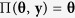. Additionally, in order to assess the global variability evolution of the posterior under different conditions, we computed the ratio of global variances between the prior and *i* posterior training sets as 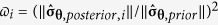.

### Control structure of the network

Flux control coefficients 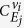 quantify the amount of control exerted by each reaction on the network. Mathematically, these coefficients represent the percentage change in the observable flux *i* per unit percent change in the enzyme *j* concentration level.


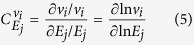


The flux control coefficient of an enzyme is a system property and their validity is restricted to the chosen reference state. Numerical computation of the above coefficients was achieved using a central finite difference scheme with a step size equal to 0.01% perturbation on the nominal *E*_*j*_ value of 1, which ensured low truncation error and avoided sensitivity to simulation error. The validity of this approximation was tested by imposing a maximum 1% deviation from the summation theorem^70^. The predictive posterior expectancy of the flux control coefficients was computed then using Eq. (4) by setting 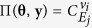.

### Model selection: identifying missing allosteric interactions

For two competing models *m*_A_ and *m*_B_, we want to choose the most likely model explaining the data **y** using Bayesian model selection. If the competing models are considered equally probable (i.e., uniform prior), the evidence in favour of *m*_A_ over *m*_B_ is computed as follows^30^,


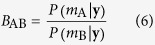


where *P*(*m*_A_|**y**) and *P*(*m*_B_|**y**) represent respectively the posterior marginal probability for model A and B, and *B*_AB_ represents the corresponding Bayes factor. Higher *B*_AB_ suggests greater support for *m*_A_ over *m*_B_. Model selection within the ABC rejection sampler was performed as described by Grelaud *et al*.^71^. Briefly, model selection is performed by accepting particles from different model structures that explain the data. To provide a fair evaluation, the index of the model to be tested is randomly sampled before the conventional rejection step takes place. Once enough particles have been accepted, the relative number of accepted instances for each structure represents the respective marginal posterior probability, and *B*_AB_ can be computed directly as the ratio of the number of accepted particles. Notably, Bayesian model selection implicitly penalizes structure complexity, as higher dimensional structures have lower probabilities of acceptance unless they significantly improve the fit. In our particular case, we employed model selection to determine the most likely allosteric interactions to be added to an incomplete metabolic model. Exploration of this space was performed by adding one interaction at a time. Significant interactions were selected based on the evidence in favour over the incomplete model. Interactions with a Bayes factor greater than three were chosen as significant interactions (refer to Supplementary Table S12 for interpretation).

### Gibbs free energy of reaction ranges

Feasible ranges for the Gibbs free energy of reaction (Δ*G*_r_) were calculated employing a modified version of Thermodynamic Variability Analysis (TVA)^72^. In standard TVA, the activity of each metabolite ln(*x*) and the Δ*G*_r_ of each reaction are minimized and maximized subject to thermodynamic and mass balance constraints, rendering a Mixed-Integer Linear Programming problem (MILP). However, in our case the flux directions are fixed as the reference flux distribution **v**^ref^ is known. Thus, an equivalent simpler LP formulation was used instead with continuous variables Δ**G**_r_ and ln(**x**),


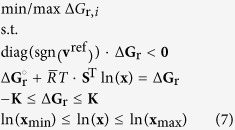


Here, **K** represents a vector with sufficiently large equal components (for example 10^3^), **x**_min_ and **x**_max_ denote the minimum and maximum concentrations bounds, **S** represents the stoichiometric matrix, 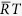 is the product of the universal gas constant and the absolute temperature and 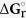 is the standard Gibbs free energy of reaction (Supplementary Table S3). The latter quantities were estimated at 37 °C, pH 7 and 0.15 ionic strength using eQuilibrator^73^. The minimum and maximum concentrations used in these calculations can be found in the Supplementary Table S4.

### Computation and implementation

The presented computational framework was implemented in the MATLAB 2015a environment (The MathWorks, Natick, MA). Given the convenient structure of the proposed workflow, accelerated convergence was achieved by dividing and distributing jobs using the MATLAB Parallel Computing Toolbox. On average, convergence of the ABC algorithm was obtained after 13–22 h for 10^3^ accepted parameter sets. Parameterization and sampling of the metabolic reactions was performed using GRASP^28^. For the estimation of the feasible Gibbs free energy ranges, we employed the LP solver *linprog* contained in the MATLAB Optimization Toolbox. Execution of the ABC sampler was performed on a 16-CPU 64-GB ram Virtual Machine hosted in the QRIScloud Polaris cell. *A posteriori* analyses of the sampling results were executed on a Dell OptiPlex 760 Desktop (Intel Core 2 Duo processor, 4 GB ram, Microsoft Windows 7, x86-based architecture).

## Additional Information

**How to cite this article**: Saa, P. A. and Nielsen, L. K. Construction of feasible and accurate kinetic models of metabolism: A Bayesian approach. *Sci. Rep*. **6**, 29635; doi: 10.1038/srep29635 (2016).

## Supplementary Material

Supplementary Information
