# Supplementary material for: Construction of feasible and accurate kinetic models of metabolism: A Bayesian approach
Source: Sci Rep. 2016 Jul 15;6:29635. doi: 10.1038/srep29635 (PMC4945864; doi:10.1038/srep29635)
Supplement: Supplementary Information [file srep29635-s1.doc]

**Supplementary Information**

**Construction of feasible and accurate kinetic models of metabolism: A Bayesian approach**

Pedro A. Saa^1^, Lars K. Nielsen^1*^

^1^Australian Institute for Bioengineering and Nanotechnology (AIBN), The University of Queensland, Brisbane, QLD 4072, Australia

^*^Corresponding author

Email: lars.nielsen@uq.edu.au

**Table of contents**

[Supplementary text 4](#__RefHeading___Toc452129643)

[1. Model description: Methionine cycle 4](#__RefHeading___Toc452129644)

[1.1. Methionine influx (*v*_MATI_) and folate conservation 4](#__RefHeading___Toc452129645)

[1.2. Methionine adenosyl transferase I (*v*_MATI_) 4](#__RefHeading___Toc452129646)

[1.3. Methionine adenosyl transferase III (*v*_MATIII_) 4](#__RefHeading___Toc452129647)

[1.4. Protein consumption for biosynthesis (*v*_PROT_) 5](#__RefHeading___Toc452129648)

[1.5. Functional methylases (*v*_METH_) 5](#__RefHeading___Toc452129649)

[1.6. Glycine N-methyltransferase (*v*_METH_) 5](#__RefHeading___Toc452129650)

[1.7. S-adenosylhomocysteine hydrolase (*v*_AHC_) 5](#__RefHeading___Toc452129651)

[1.8. Methionine synthase (*v*_MS_) 5](#__RefHeading___Toc452129652)

[1.9. Betaine-homocysteine methyltransferase (*v*_BHMT_) 6](#__RefHeading___Toc452129653)

[1.10. Cystathionine beta-synthase (*v*_CBS_) 6](#__RefHeading___Toc452129654)

[1.11. Methylenetetrahydrofolate reductase (*v*_MTHFR_) 6](#__RefHeading___Toc452129655)

[Supplementary figures 7](#__RefHeading___Toc452129656)

[Figure S1. Parameter convergence and posterior predictions differing the order of dataset addition. 7](#__RefHeading___Toc452129657)

[Figure S2. Boxplots of the posterior distribution of selected parameters as a function of the dataset size. 8](#__RefHeading___Toc452129658)

[Figure S3. Qualitative assessment of the control structure prediction. 9](#__RefHeading___Toc452129659)

[Figure S4. Stability assessment of the posterior sample under different reference conditions. 10](#__RefHeading___Toc452129660)

[Figure S5. Predictive posterior predictions for the 12 validation perturbations using the posterior trained with dataset #2. 11](#__RefHeading___Toc452129661)

[Figure S6. Analysis of compensatory effects in the *GNMT* reaction. 12](#__RefHeading___Toc452129662)

[Figure S7. Analysis of the sample size impact on selected summary statistics of the sampled parameters. 13](#__RefHeading___Toc452129663)

[Supplementary tables 14](#__RefHeading___Toc452129664)

[Table S1. Description of the training dataset for the model construction 14](#__RefHeading___Toc452129665)

[Table S2. Kinetic description of each enzyme in the network 15](#__RefHeading___Toc452129666)

[Table S3. Estimated minimum and maximum $\Delta G_{\text{r}}^{\text{ref}}$ for the methionine cycle 16](#__RefHeading___Toc452129667)

[Table S4. Minimum and maximum concentrations employed in the calculation of $\Delta G_{\text{r}}^{\text{ref}}$ 17](#__RefHeading___Toc452129668)

[Table S5. Validation set of flux responses in mmol/L-cells/h under different genetic conditions 18](#__RefHeading___Toc452129669)

[Table S6. Comparison of the average expected root-mean-square error (mmol/L-cells/h) for different validation perturbations between posterior predictions using dataset #2 and the full training set 19](#__RefHeading___Toc452129670)

[Table S7. Simulated flux distributions in mmol/L-cells/h for different genetic perturbations assuming various levels of experimental noise 20](#__RefHeading___Toc452129671)

[Table S8. Error statistics comparison of the root-mean-square flux calibration error for the base case, the case with 10% and 20% experimental noise addition 21](#__RefHeading___Toc452129672)

[Table S9. Model selection results for the addition of the first interaction 22](#__RefHeading___Toc452129673)

[Table S10. Model selection results for the addition of the second interaction 23](#__RefHeading___Toc452129674)

[Table S11. Marginal probabilities for similar model structures 24](#__RefHeading___Toc452129675)

[Table S12. Bayes factor interpretation adapted from Kass & Raftery^49^ 25](#__RefHeading___Toc452129676)

Supplementary text

1. Model description: Methionine cycle

The methionine model comprises a system of five ordinary differential equations and one algebraic equation (folate pool conservation) describing intracellular concentrations (in μM) of the main intermediates involved in the cycle, namely: methionine (Met), S-adenosylmethionine (AdoMet), S-adenosylhomocysteine (AdoHcy), homocysteine (Hcy), methyl-tetrahydrofolate (5-CH_3_-THF) and 5,10-methylenetetrahydrofolate (5,10-CH_2_-THF) (Eqs. S1). The mathematical description of each reaction (in mmol/h/L-cells) is based on the most experimentally-supported representation of this system to date[^1^](#_ENREF_1), and reflects over a decade of work on this pathway[^2-6^](#_ENREF_2). The following subsections describe in detailed the parameterization employed for each reaction.

$\begin{matrix} \text{X}_{\text{biomass}}^{-1}\cdot\frac{d\left[\text{Met}\right]}{dt}=v_{\text{INFLUX}}+v_{\text{BHMT}}+v_{\text{MS}}-(v_{\text{PROT}}+v_{\text{MATI}}+v_{\text{MATIII}}) \\ \text{X}_{\text{biomass}}^{-1}\cdot\frac{d\left[\text{AdoMet}\right]}{dt}=v_{\text{MATI}}+v_{\text{MATIII}}-(v_{\text{METH}}+v_{\text{GNMT}}) \\ \text{X}_{\text{biomass}}^{-1}\cdot\frac{d\left[\text{AdoHcy}\right]}{dt}=v_{\text{METH}}+v_{\text{GNMT}}-v_{\text{AHC}} \\ \text{X}_{\text{biomass}}^{-1}\cdot\frac{d\left[\text{Hcy}\right]}{dt}=v_{\text{AHC}}-(v_{\text{BHMT}}+v_{\text{MS}}+v_{\text{CBS}}) \\ \text{X}_{\text{biomass}}^{-1}\cdot\frac{d\text{[5-CH}_{3}\text{-THF]}}{dt}=v_{\text{MTHFR}}-v_{\text{MS}} \\ r\cdot\text{[5,10-CH}_{2}\text{-THF]+[5-CH}_{3}\text{-THF]}=[\text{Folate]}_{\text{pool}} \end{matrix}$ (S1)

- 1. Methionine influx (*v*_MATI_) and folate conservation

The methionine uptake flux (*v*_INFLUX_) was set to 0.76 mmol/h/L-cells in agreement with recent observations[^7^](#_ENREF_7). In the case of folates conservation, the pool size [Folate]_pool_ was set to 25 (μM) based on experimental observations in rats[^8-10^](#_ENREF_8). The factor r was set to 5 to account for the total pool of total concentration of all intracellular folates except dihydrofolate (DHF, inactive form of tetrahydrofolate THF) as reported by Korendyaseva et al.[^1^](#_ENREF_1).

- 1. Methionine adenosyl transferase I (*v*_MATI_)

Equation S2 describes the dependency of *v*_MATI_ of [Met] and the competitive inhibition by [AdoMet][^2^](#_ENREF_2).

$v_{^{\text{MATI}}}=\frac{2.2}{1+25\left(1+\text{[AdoMet]}/25\right)/\text{[Met]}}$ (S2)

- 1. Methionine adenosyl transferase III (*v*_MATIII_)

MATIII kinetics was modelled using the MWC model[^11^](#_ENREF_11). This rate includes positive cooperative dependence on methionine as well as activation[^12^](#_ENREF_12) and inhibition at different [AdoMet][^13^](#_ENREF_13). Detailed derivation of the reaction rate is provided elsewhere[^1^](#_ENREF_1).

$\begin{matrix} v_{\text{MATIII}}=A^{\text{MATIII}}\cdot\frac{[\text{Met}]^{2}+K_{\text{S,eff}}^{\text{MATIII}}\cdot\beta\cdot[\text{Met}]}{[\text{Met}]^{2}+2\cdot K_{\text{S,eff}}^{\text{MATIII}}\cdot\beta\cdot[\text{Met}]+\left(K_{\text{S,eff}}^{\text{MATIII}}\right)^{2}} \\ A^{\text{MATIII}}=\frac{15.8}{1+[\text{AdoMet}]/700},\text{ }K_{\text{S,eff}}^{\text{MATIII}}=\frac{1200}{1+[\text{AdoMet}]/50}\text{ , }\beta=0.1 \end{matrix}$ (S3)

- 1. Protein consumption for biosynthesis (*v*_PROT_)

The rate of methionine consumption in protein turnover can be phenomenologically described a by simple Michaelis-Menten kinetics[^1^](#_ENREF_1). The latter actually assumes that the first step of protein synthesis, i.e., charging of Met-tRNA catalyzed by methionyl-tRNA-synthetase, can be modelled with this expression[^14^](#_ENREF_14).

$v_{\text{PROT}}=\frac{0.188\cdot[\text{Met}]}{[\text{Met}]+18}$ (S4)

- 1. Functional methylases (*v*_METH_)

There is a great diversity of intracellular methylases (apart from GNMT) catalysing methyl group transfer from AdoMet to different methyl group acceptors. To describe this kinetics, a Michaelis-Menten type kinetics has been proposed including product inhibition by AdoHyc[^2^](#_ENREF_2).

$v_{\text{METH}}=\frac{13}{1+\frac{1+0.25\cdot[\text{AdoHcy}]}{[\text{AdoMet}]}+10\cdot\left(1+\frac{\left(1+0.25\cdot[\text{AdoHcy]}\right)}{[\text{AdoMet}]}\right)}$ (S5)

- 1. Glycine N-methyltransferase (*v*_METH_)

This expression describes cooperative behaviour upon binding AdoMet with *n*_Hill_ = 2.3[^15^](#_ENREF_15), and it also includes competitive inhibition by AdoHcy[^16^](#_ENREF_16) and non-competitive inhibition by 5,10-CH_3_-THF[^17^](#_ENREF_17). The kinetics is represented by the following mathematical expression[^1^](#_ENREF_1),

$v_{\text{GNMT}}=\frac{60}{1+\left(\frac{350\cdot\left(1+[\text{AdoHcy}]/52\right)}{[\text{AdoMet}]}\right)^{2.3}}\cdot\frac{1}{1+[5,10\text{-CH}_{3}\text{-THF}]/0.3}$ (S6)

- 1. S-adenosylhomocysteine hydrolase (*v*_AHC_)

This enzyme has a relatively high activity in hepatocytes which greatly exceeds the activities of the other enzymes involved in methionine metabolism. To describe this behaviour, Korendyaseva et al.[^1^](#_ENREF_1) have employed an equilibrium assumption. Here, we have opted for a more realistic expression based on a rapid equilibrium instead[^6^](#_ENREF_6).

$v_{\text{AHC}}=100\cdot\left([\text{AdoHcy}]-10\cdot[\text{Hcy}]\right)$ (S7)

- 1. Methionine synthase (*v*_MS_)

An irreversible Michaelis-Menten type kinetics was employed to describe the kinetics of this enzyme[^1^](#_ENREF_1).

$v_{\text{MS}}=\frac{6.6}{1+\frac{89}{[5,10\text{-CH}_{2}\text{-THF}]}+\frac{1}{[\text{Hcy}]}\cdot\left(1+\frac{142}{[5,10\text{-CH}_{2}\text{-THF}]}\right)}$ (S8)

- 1. Betaine-homocysteine methyltransferase (*v*_BHMT_)

This enzyme catalyses an irreversible reaction and displays inhibition by its products methionine and dimethyglycine[^18^](#_ENREF_18). As the concentration of dimethyglycine is considered to be constant, the reaction rate can be described by the following expression[^1^](#_ENREF_1).

$v_{\text{BHMT}}=\frac{60}{51+\frac{12}{[\text{Hcy}]}\cdot\left(1+\frac{[\text{Met}]}{1.5}\right)}$ (S9)

- 1. Cystathionine beta-synthase (*v*_CBS_)

The rate for this enzyme describes the dependence of the velocity of reaction on its substrates and includes activation by AdoMet[^5^](#_ENREF_5).

$v_{\text{CBS}}=\frac{121}{5.24+1001.7/[\text{Hcy}]}\cdot\left(1+\frac{1}{1+60/[\text{AdoMet}]}\right)$ (S10)

- 1. Methylenetetrahydrofolate reductase (*v*_MTHFR_)

MTHFR exhibits a complex kinetic regulation with AdoMet as an allosteric inhibitor and AdoHyc as an activator. The following expression describes the above mechanism[^1^](#_ENREF_1).

$\begin{matrix} v_{\text{MTHFR}}=\frac{A^{\text{MTHFR}}}{1+0.08\cdot\left(1+\frac{[5\text{-CH}_{3}\text{-THF}]}{20}\right)+\frac{88}{[5,10\text{-CH}_{2}\text{-THF}]}} \\ A^{\text{MTHFR}}=4.08\cdot\left(\frac{1}{1+[\text{AdoMet}]/K_{\text{i,AdoMet}}^{\text{MTHFR}}}+\frac{0.046}{1+K_{\text{i,AdoMet}}^{\text{MTHFR}}/[\text{AdoMet}]}\right) \\ K_{\text{i,AdoMet}}^{\text{MTHFR}}=3\cdot\left(1+[\text{AdoHcy}]/3\right) \end{matrix}$ (S11)

Supplementary figures

**A**

**B**

Figure S1. Parameter convergence and posterior predictions differing the order of dataset addition.

Panel A) Results support dataset #2 as the most informative of the first three datasets. These results also indicate consistent convergence of the parameter posterior distribution to a specific a region of the parameter space. Panel B) Expected posterior predictions do not depend on the order of addition of the data.

Figure S2. Boxplots of the posterior distribution of selected parameters as a function of the dataset size.


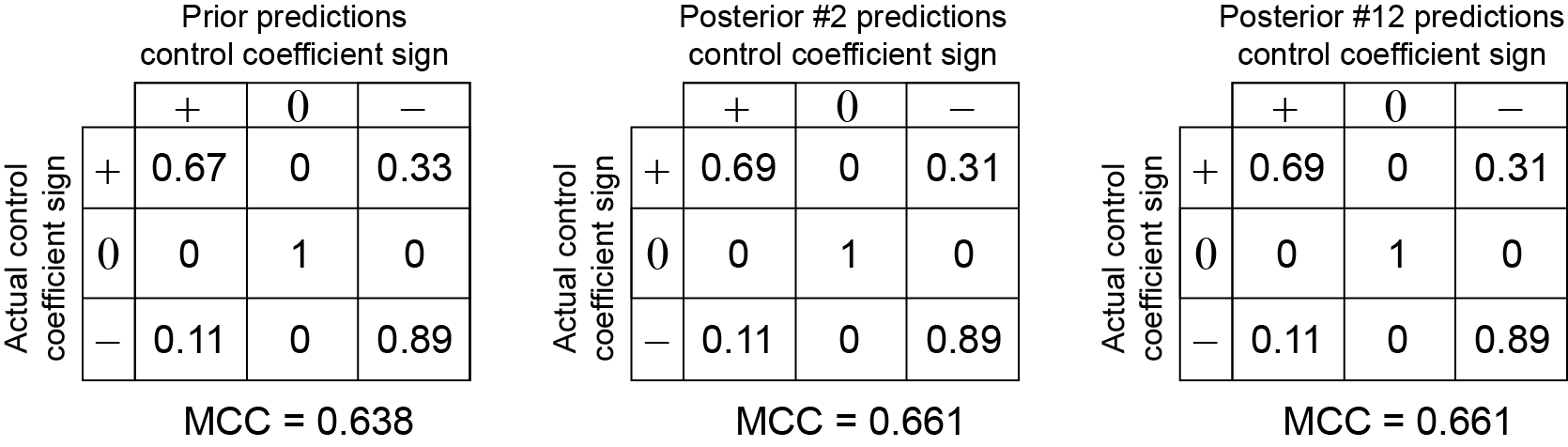


Figure S3. Qualitative assessment of the control structure prediction.

The confusion matrix describes the accuracy in the prediction of flux control coefficient signs using the prior and posteriors derived from dataset #2 and dataset #12. There is surprisingly a good agreement between all the predictions and the actual flux control coefficients signs at the reference point as observed by the high Matthew’s correlation coefficient (MCC) (MCCmax = 1) as defined by Gorodkin[^21^](#_ENREF_21) for multi-class prediction assessment.

Figure S4. Stability assessment of the posterior sample under different reference conditions.

For different conditions (reference state, dataset #2 and dataset #12) all the samples are stable, i.e., Jacobians do not have a positive real eigenvalue max(Re(λ)) = λ_max_ < 0. We also note that in most cases, the minimum eigenvalue is fairly negative (relative to the λ_max_) which also suggests a robust stable response of the system.

Figure S5. Predictive posterior predictions for the 12 validation perturbations using the posterior trained with dataset #2.

Boxplots represent the posterior predictive distribution whereas the red circles describe the true model responses. There is overall good agreement between predicted and true model responses, although slightly worse than in the case where the full training set was used (refer to Table S5 for more details).


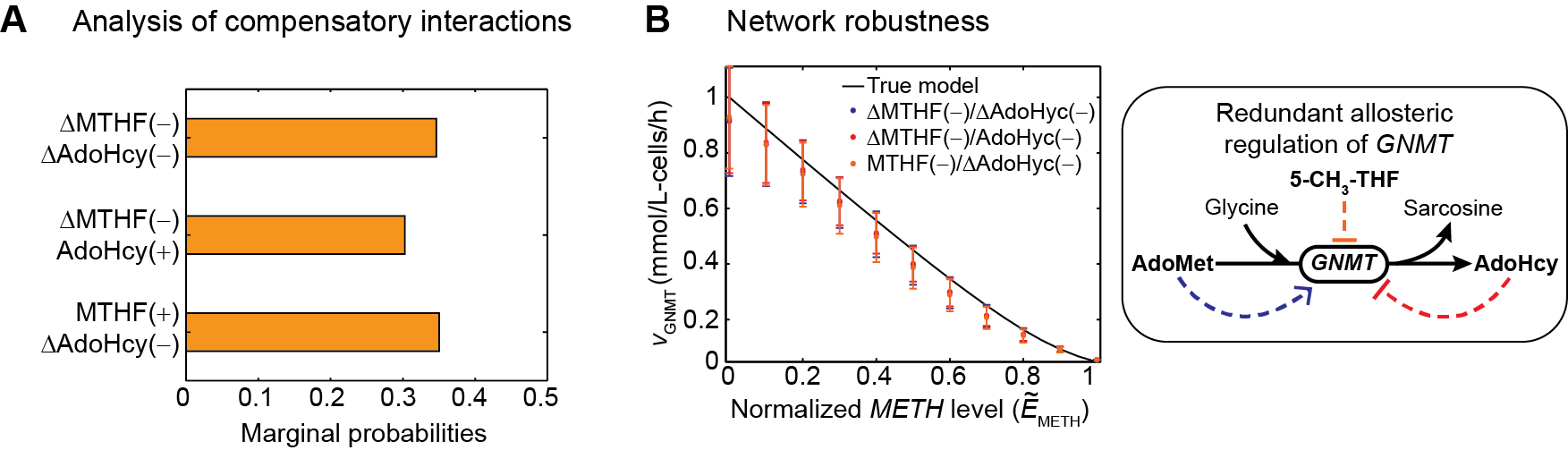


Figure S6. Analysis of compensatory effects in the *GNMT* reaction.

A Model selection was performed to test three alternative regulatory structures affecting *GNMT*.
Structure 1 lacks both the 5-CH_3_-THF and AdoHyc interactions (∆5-CH_3_-THF, ∆AdoHcy). Structure 2 lacks only the 5-CH_3_-THF interaction (∆5-CH_3_-THF), whereas Structure 3 only lacks the AdoHyc interaction (∆AdoHcy). Analysis of the marginal probabilities for each model structure suggests no significant positive influence of any model structure.

B Multiple allosteric effectors exert very similar kinetic responses of *GNMT*. The homotropic interaction by AdoMet and the heterotropic interactions by AdoHyc and 5-CH_3_-THF are equally capable of regulating the activity of *GNMT* upon down-regulation of *METH* (parallel reaction). This regulatory redundancy points to network robustness. In this case, more evidence (simulated data) will be needed to determine the correct structure.

Figure S7. Analysis of the sample size impact on selected summary statistics of the sampled parameters.

For different sample sizes *N* = 10^3^, 2∙10^3^ and 5∙10^3^, there is good agreement between the main summary statistics (mean and covariance) of the sampled parameters (in all cases, correlation > 0.99).

Supplementary tables

Table S1. Description of the training dataset for the model construction

| Reaction | Reaction flux (mmol/L-cells/h) | | | | | | | | | | | |
| --- | --- | --- | --- | --- | --- | --- | --- | --- | --- | --- | --- | --- |
|  | dataset #1 | dataset #2 | dataset #3 | dataset #4 | dataset #5 | dataset #6 | dataset #7 | dataset #8 | dataset #9 | dataset #10 | dataset #11 | dataset #12 |
| *v*_INFLUX_ | 0.761 | 1.142 | 0.761 | 0.761 | 0.761 | 0.761 | 0.761 | 0.761 | 0.761 | 0.761 | 0.761 | 0.761 |
| *v*_PROT_ | 0.133 | 0.148 | 0.108 | 0.135 | 0.138 | 0.138 | 0.201 | 0.153 | 0.138 | 0.132 | 0.139 | 0.142 |
| *v*_MATI_ | 0.869 | 0.615 | 0.730 | 0.856 | 0.836 | 0.815 | 0.837 | 0.591 | 0.843 | 0.680 | 0.612 | 0.736 |
| *v*_MATIII_ | 0.158 | 0.815 | 0.058 | 0.184 | 0.238 | 0.249 | 0.182 | 0.384 | 0.232 | 0.426 | 0.401 | 0.376 |
| *v*_METH_ | 1.004 | 1.082 | 0.785 | 0.969 | 1.006 | 1.016 | 0.991 | 0.956 | 1.001 | 1.036 | 0.763 | 1.026 |
| *v*_GNMT_ | 0.023 | 0.349 | 0.003 | 0.071 | 0.068 | 0.048 | 0.027 | 0.018 | 0.073 | 0.069 | 0.250 | 0.085 |
| *v*_AHC_ | 1.027 | 1.431 | 0.788 | 1.040 | 1.074 | 1.064 | 1.019 | 0.974 | 1.074 | 1.105 | 1.013 | 1.111 |
| *v*_MS_ | 0.088 | 0.063 | 0.134 | 0.021 | 0.091 | 0.085 | 0.092 | 0.102 | 0.090 | 0.079 | 0.059 | 0.083 |
| *v*_BHMT_ | 0.310 | 0.374 | 0.000 | 0.392 | 0.359 | 0.356 | 0.366 | 0.264 | 0.361 | 0.397 | 0.331 | 0.409 |
| *v*_CBS_ | 0.629 | 0.994 | 0.654 | 0.627 | 0.623 | 0.623 | 0.560 | 0.608 | 0.623 | 0.630 | 0.623 | 0.620 |
| *v*_MTHFR_ | 0.088 | 0.063 | 0.134 | 0.021 | 0.091 | 0.085 | 0.092 | 0.102 | 0.090 | 0.079 | 0.059 | 0.083 |
| Enzyme | Enzymatic expression relative to the reference state | | | | | | | | | | | |
| *INFLUX* | 1.0 | 1.5 | 1.0 | 1.0 | 1.0 | 1.0 | 1.0 | 1.0 | 1.0 | 1.0 | 1.0 | 1.0 |
| *PROT* | 1.0 | 1.0 | 1.0 | 1.0 | 1.0 | 1.0 | 1.5 | 1.0 | 1.0 | 1.0 | 1.0 | 1.0 |
| *MATI* | 1.0 | 1.0 | 1.0 | 1.0 | 1.0 | 1.0 | 1.0 | 0.5 | 1.0 | 1.0 | 1.0 | 1.0 |
| *MATIII* | 1.0 | 1.0 | 1.0 | 1.0 | 1.0 | 1.0 | 1.0 | 1.0 | 1.0 | 2.0 | 1.0 | 1.0 |
| *METH* | 1.0 | 1.0 | 1.0 | 1.0 | 1.0 | 1.0 | 1.0 | 1.0 | 1.0 | 1.0 | 0.7 | 1.0 |
| *GNMT* | 1.0 | 1.0 | 1.0 | 1.0 | 1.0 | 1.0 | 1.0 | 1.0 | 2.0 | 1.0 | 1.0 | 1.0 |
| *AHC* | 1.0 | 1.0 | 1.0 | 1.0 | 1.0 | 2.0 | 1.0 | 1.0 | 1.0 | 1.0 | 1.0 | 1.0 |
| *MS* | 1.0 | 1.0 | 1.0 | 1.0 | 2.0 | 1.0 | 1.0 | 1.0 | 1.0 | 1.0 | 1.0 | 1.0 |
| *BHMT* | 1.0 | 1.0 | 0.0 | 1.0 | 1.0 | 1.0 | 1.0 | 1.0 | 1.0 | 1.0 | 1.0 | 1.0 |
| *CBS* | 1.5 | 1.0 | 1.0 | 1.0 | 1.0 | 1.0 | 1.0 | 1.0 | 1.0 | 1.0 | 1.0 | 0.7 |
| *MTHFR* | 1.0 | 1.0 | 1.0 | 0.2 | 1.0 | 1.0 | 1.0 | 1.0 | 1.0 | 1.0 | 1.0 | 1.0 |

Table S2. Kinetic description of each enzyme in the network

| Reaction | EC Number | Catalytic mechanism | Kinetic description | References |
| --- | --- | --- | --- | --- |
| *v*_INFLUX_ | - | Fixed rate | Methionine intake | - |
| *v*_PROT_ | - | Ordered Uni-Uni | Phenomenological description of methionine consumption | Korendyaseva et al.[^1^](#_ENREF_1) |
| *v*_MATI_ | 2.5.1.6 | Ordered Bi-Bi | Competitive inhibition by AdoMet. The mechanism is consistent with the following sequential mechanism: i) formation of an E·ATP·Met complex; ii) reaction to form the compulsory intermediate E·AdoMet·PPPi complex; and, iii) subsequent hydrolysis of PPPi before the regeneration of the enzyme by product release (AdoMet) | Markham & Pajares[^22^](#_ENREF_22) |
| *v*_MATIII_ | 2.5.1.6 | Ordered Bi-Bi | Same catalytic mechanism as MATI, however this isoform displays allosteric activation by AdoMet. This enzyme has two identical catalytic subunits (homodimer). | Markham & Pajares[^22^](#_ENREF_22) |
| *v*_METH_^a^ | 2.1.1.12 | Ordered Bi-Bi | AdoMet binds first and AdoHcy is released last. | James et al.[^23^](#_ENREF_23) |
| *v*_GNMT_ | 2.1.1.20 | Ordered Bi-Bi | This enzyme has four subunits and displays cooperative binding for AdoMet. Two negative effectors have been described so far; AdoHcy and 5,10-CH_3_-THF. The most likely catalytic mechanism involves AdoMet and AdoHcy binding and releasing first from the enzyme, respectively. | Huang et al.[^24^](#_ENREF_24); Takata et al.[^25^](#_ENREF_25); Yeo et al.[^17^](#_ENREF_17) |
| *v*_AHC_ | 3.3.1.1 | Ordered Bi-Bi | AdoHcy and Hcy are the first substrate and product to bind and release from the enzyme. This enzyme is tetrameric and requires NAD^+^ to maintain its quaternary structure and catalytic activity. | Tehlivets et al.[^26^](#_ENREF_26) |
| *v*_MS_ | 2.1.1.13 | Ordered Bi-Bi | 5-CH_3_-THF binds first and Met is the first product of the reaction. | Chen et al.[^27^](#_ENREF_27) |
| *v*_BHMT_ | 2.1.1.5 | Ordered Bi-Bi | The catalytic mechanism is consistent with Hcy binding first and dimethilglycine as the first product released. | Finkelstein et al.[^18^](#_ENREF_18) |
| *v*_CBS_ | 4.2.1.22 | Ordered Bi-Bi | The enzyme follows a compulsory-order mechanism with serine binding first and cystathionine as the first released product. | Borcsok & Abeles[^28^](#_ENREF_28) |
| *v*_MTHFR_ | 1.5.1.20 | Ping-Pong | Allosteric effectors for the dimeric enzyme are AdoMet (negative) and AdoHcy (positive). The catalytic mechanism is consistent with NADPH binding first and 5-CH_3_-THF as the second product released. | Matthews & Daubner[^20^](#_ENREF_20); Jencks & Matthews[^19^](#_ENREF_19); Trimmer et al.[^29^](#_ENREF_29); Vanoni & Matthews[^30^](#_ENREF_30) |

^a^There are several enzymes capable of performing the transfer of the methyl group from S-adenosyl-methionine to a different compound. Here, we have regarded the methionine methyltransferase reaction (E.C. 2.1.1.12) as a good representative if this group based on the well-known thermodynamics and kinetic information.

Table S3. Estimated minimum and maximum $\Delta G_{\text{r}}^{\text{ref}}$ for the methionine cycle

| Reaction | Stoichiometry | $\Delta G_{\text{r}}^{\text{o}}$ (kJ/mol)^a^ | $\Delta G_{\text{r,min}}^{'}$ (kJ/mol) | $\Delta G_{\text{r,max}}^{'}$ (kJ/mol) |
| --- | --- | --- | --- | --- |
| *v*_INFLUX_^b^ | → met | - | - | - |
| *v*_PROT_^b^ | met → prot | - | - | - |
| *v*_MATI/III_ | h_2_o + atp + L-methionine → orthophosphate + diphosphate + S-adenosyl-L-methionine | -77.0 | -112.5 | -90.8 |
| *v*_METH_ | S-adenosyl-L-methionine + group acceptor → S-adenosyl-L-homocysteine + methyl-group acceptor | -1.0 | -22.4 | 0.0 |
| *v*_GNMT_ | S-adenosyl-L-methionine + glycine → S-adenosyl-L-homocysteine + sarcosine | 10.4 | -29.6 | -7.7 |
| *v*_AHC_ | S-adenosyl-L-homocysteine + h_2_o → L-homocysteine + adenosine | 34.8 | -18.1 | 0.0 |
| *v*_MS_ | L-homocysteine + 5-methyltetrahydrofolate → L-methionine + tetrahydrofolate | -10.4 | -7.1 | 0.0 |
| *v*_BHMT_ | L-homocysteine + betaine → L-methionine + N,N-dimethylglycine | -4.8 | -18.2 | 0.0 |
| *v*_CBS_ | L-serine + L-homocysteine → cystathionine + h_2_o | -30.1 | -8.9 | 0.0 |
| *v*_MTHFR_ | nadph + 5,10-methylenetetrahydrofolate → nadp + 5-methyltetrahydrofolate + h_2_o | -38.2 | -57.5 | -43.5 |

^a^Standard conditions were defined at pH = 7, ionic strength = 0.15 and T = 298.15 K. Calculation of these quantities was performed using eQuilibrator[^31^](#_ENREF_31).

^b^A fixed thermodynamic affinity of -100 (kJ/mol) (i.e., irreversible) was used for these reactions to determine the feasible Gibbs free energy ranges during TVA.

Table S4. Minimum and maximum concentrations employed in the calculation of $\Delta G_{\text{r}}^{\text{ref}}$

| Metabolite | Minimum (M) | Maximum (M) | Reference |
| --- | --- | --- | --- |
| L-methionine | 2·10^-5^ | 7.5·10^-5^ | Finkelstein et al.[^32^](#_ENREF_32); Finkelstein & Martin[^33^](#_ENREF_33); Jacobs et al.[^34^](#_ENREF_34) |
| atp | 10^-3^ | 2·10^-3^ | Chen et al.[^35^](#_ENREF_35) |
| h_2_o | 50 | 50 | Wolfe[^36^](#_ENREF_36) |
| S-adenosyl-L-methionine | 5·10^-5^ | 1.7·10^-4^ | Finkelstein et al.[^32^](#_ENREF_32); Finkelstein & Martin[^33^](#_ENREF_33) |
| diphosphate^a^ | 10^-4^ | 2.5·10^-3^ | Kukko and Heinonen[^37^](#_ENREF_37) |
| orthophosphate | 10^-3^ | 10^-3^ | Bevington et al.[^38^](#_ENREF_38) |
| group acceptor^b^ | 10^-4^ | 10^-2^ |  |
| S-adenosyl-L-homocysteine | 3·10^-6^ | 4·10^-5^ | Finkelstein et al.[^32^](#_ENREF_32); Finkelstein & Martin[^33^](#_ENREF_33) |
| methyl-group acceptor^b^ | 10^-4^ | 10^-2^ |  |
| sarcosine^b^ | 10^-8^ | 10^-6^ |  |
| glycine | 1.2·10^-3^ | 1.8·10^-3^ | Regina et al.[^39^](#_ENREF_39); Scheer et al.[^40^](#_ENREF_40) |
| L-homocysteine | 3·10^-6^ | 6·10^-6^ | Vivitsky et al.[^41^](#_ENREF_41) |
| adenosine | 4·10^-7^ | 5·10^-5^ | Bontemps et al.[^42^](#_ENREF_42); Chagoya et al.[^43^](#_ENREF_43) |
| 5-methyltetrahydrofolate | 10^-6^ | 1.6·10^-5^ | Horne[^10^](#_ENREF_10); Ozias & Schalinske[^9^](#_ENREF_9); Taes et al.[^8^](#_ENREF_8) |
| 5,10-methylenetetrahydrofolate | 10^-6^ | 8·10^-6^ | Horne[^10^](#_ENREF_10); Ozias & Schalinske[^9^](#_ENREF_9); Taes et al.[^8^](#_ENREF_8) |
| betaine | 2.2·10^-4^ | 7.3·10^-3^ | Finkelstein & Martin[^33^](#_ENREF_33); Garrow et al.[^44^](#_ENREF_44); Millian et al.[^45^](#_ENREF_45) |
| N,N-dimethylglycine | 10^-5^ | 2.5·10^-4^ | Garrow et al.[^44^](#_ENREF_44) |
| L-serine | 1.4·10^-4^ | 1.8·10^-3^ | Finkelstein & Martin[^33^](#_ENREF_33) |
| cystathionine | 10^-6^ | 5·10^-6^ | Singh et al.[^46^](#_ENREF_46) |
| nadph | 2·10^-4^ | 3·10^-4^ | Jencks & Matthews[^19^](#_ENREF_19); Vanoni & Matthews[^30^](#_ENREF_30) |
| nadp | 10^-6^ | 1.5·10^-6^ | Veech et al.[^47^](#_ENREF_47) |
| tetrahydrofolate | 1.8·10^-5^ | 2.8·10^-5^ | Horne[^10^](#_ENREF_10) |

^a^Values estimated from *E. coli* data.

^b^These values were arbitrarily set based on the calculation of the feasible ranges for $\Delta G_{\text{r}}^{\text{ref}}$.

Table S5. Validation set of flux responses in mmol/L-cells/h under different genetic conditions

| Reaction | –50% CBS | –50% *v*_INFLUX_ | 2X BHMT | +80% MTHFR | –50% MS | –50% AHC | –50% *v*_PROT_ | +50% MATI | –50% GNMT | –50% MATIII | +30% METH | +30% CBS |
| --- | --- | --- | --- | --- | --- | --- | --- | --- | --- | --- | --- | --- |
| *v*_INFLUX_ | 0.76 | 0.38 | 0.76 | 0.76 | 0.76 | 0.76 | 0.76 | 0.76 | 0.76 | 0.76 | 0.76 | 0.76 |
| *v*_PROT_ | 0.14 | 0.10 | 0.15 | 0.14 | 0.14 | 0.14 | 0.07 | 0.13 | 0.14 | 0.14 | 0.14 | 0.13 |
| *v*_MATI_ | 0.63 | 0.78 | 0.76 | 0.78 | 0.80 | 0.81 | 0.78 | 0.93 | 0.79 | 0.90 | 1.00 | 0.85 |
| *v*_MATIII_ | 0.55 | 0.04 | 0.49 | 0.30 | 0.26 | 0.25 | 0.34 | 0.20 | 0.26 | 0.14 | 0.15 | 0.18 |
| *v*_METH_ | 1.03 | 0.82 | 1.08 | 1.04 | 1.02 | 1.02 | 1.04 | 1.04 | 1.03 | 1.00 | 1.14 | 1.01 |
| *v*_GNMT_ | 0.14 | 0.00 | 0.17 | 0.05 | 0.03 | 0.05 | 0.08 | 0.08 | 0.03 | 0.04 | 0.01 | 0.03 |
| *v*_AHC_ | 1.18 | 0.82 | 1.25 | 1.09 | 1.05 | 1.06 | 1.12 | 1.13 | 1.06 | 1.04 | 1.15 | 1.04 |
| *v*_MS_ | 0.08 | 0.12 | 0.06 | 0.13 | 0.08 | 0.09 | 0.08 | 0.08 | 0.08 | 0.09 | 0.12 | 0.09 |
| *v*_BHMT_ | 0.48 | 0.42 | 0.57 | 0.34 | 0.35 | 0.36 | 0.35 | 0.42 | 0.35 | 0.33 | 0.40 | 0.32 |
| *v*_CBS_ | 0.62 | 0.28 | 0.62 | 0.62 | 0.62 | 0.62 | 0.69 | 0.63 | 0.62 | 0.62 | 0.63 | 0.63 |
| *v*_MTHFR_ | 0.08 | 0.12 | 0.06 | 0.13 | 0.08 | 0.09 | 0.08 | 0.08 | 0.08 | 0.09 | 0.12 | 0.09 |

Table S6. Comparison of the average expected root-mean-square error (mmol/L-cells/h) for different validation perturbations between posterior predictions using dataset #2 and the full training set

| Perturbation | posterior from dataset #2 | posterior from dataset #12 | relative percentage difference |
| --- | --- | --- | --- |
| –50% CBS | 0.0714 | 0.0698 | -2.3% |
| –50% *v*_INFLUX_ | 0.1182 | 0.1135 | -4.1% |
| 2X BHMT | 0.0407 | 0.0396 | -2.8% |
| +80% MTHFR | 0.0176 | 0.0174 | -1.1% |
| –50% MS | 0.0079 | 0.0080 | 1.2% |
| –50% AHC | 0.0953 | 0.0917 | -3.9% |
| –50% *v*_PROT_ | 0.0236 | 0.0226 | -4.4% |
| +50% MATI | 0.0169 | 0.0171 | 1.2% |
| –50% GNMT | 0.0055 | 0.0054 | -1.9% |
| –50% MATIII | 0.0044 | 0.0046 | 4.3% |
| +30% METH | 0.0547 | 0.0534 | -2.4% |
| +30% CBS | 0.0244 | 0.0229 | -6.6% |

Table S7. Simulated flux distributions in mmol/L-cells/h for different genetic perturbations assuming various levels of experimental noise

| Reaction | No exp. noise added | | | 10% exp. noise added | | | 20% exp. noise added | | |
| --- | --- | --- | --- | --- | --- | --- | --- | --- | --- |
|  | Ref | +50% CBS | BHMT KO | Ref | +50% CBS | BHMT KO | Ref | +50% CBS | BHMT KO |
| *v*_INFLUX_ | 0.76 | 0.76 | 1.14 | 0.81 | 0.71 | 1.10 | 0.91 | 0.91 | 1.37 |
| *v*_PROT_ | 0.14 | 0.13 | 0.15 | 0.13 | 0.15 | 0.14 | 0.17 | 0.16 | 0.18 |
| *v*_MATI_ | 0.81 | 0.87 | 0.62 | 0.90 | 0.78 | 0.55 | 0.98 | 1.00 | 0.68 |
| *v*_MATIII_ | 0.25 | 0.16 | 0.82 | 0.27 | 0.14 | 0.80 | 0.30 | 0.13 | 0.90 |
| *v*_METH_ | 1.02 | 1.00 | 1.08 | 1.12 | 0.90 | 0.97 | 1.22 | 1.10 | 1.21 |
| *v*_GNMT_ | 0.05 | 0.02 | 0.35 | 0.05 | 0.02 | 0.38 | 0.06 | 0.02 | 0.38 |
| *v*_AHC_ | 1.06 | 1.03 | 1.43 | 1.17 | 0.92 | 1.36 | 1.28 | 1.12 | 1.58 |
| *v*_MS_ | 0.09 | 0.09 | 0.06 | 0.09 | 0.08 | 0.06 | 0.10 | 0.11 | 0.08 |
| *v*_BHMT_ | 0.36 | 0.31 | 0.37 | 0.39 | 0.28 | 0.34 | 0.43 | 0.26 | 0.32 |
| *v*_CBS_ | 0.62 | 0.63 | 0.99 | 0.69 | 0.57 | 0.96 | 0.75 | 0.75 | 1.19 |
| *v*_MTHFR_ | 0.09 | 0.09 | 0.06 | 0.09 | 0.08 | 0.06 | 0.10 | 0.11 | 0.08 |

Table S8. Error statistics comparison of the root-mean-square flux calibration error for the base case, the case with 10% and 20% experimental noise addition

| Condition | Percentile | | | |
| --- | --- | --- | --- | --- |
|  | 50% | 75% | 95% | 99% |
| Base case | 0.067 | 0.131 | 0.203 | 0.237 |
| 10% exp. noise addition | 0.064 | 0.165 | 0.226 | 0.250 |
| 20% exp. noise addition | 0.126 | 0.180 | 0.244 | 0.272 |

Table S9. Model selection results for the addition of the first interaction

| Model structure | Metabolic effector | Model instances in the posterior | Bayes factor | Lower 99%-conf. value^a^ | Upper 99%-conf. value^a^ |
| --- | --- | --- | --- | --- | --- |
| Base model | – | 21 | 1.00 | - | - |
| Base model/*v*_MATIII_ | Met(+) | 39 | 1.86 | 1.856 | 1.859 |
|  | Met(–) | 26 | 1.24 | 1.237 | 1.239 |
|  | **AdoMet(+)** | **64** | **3.05** | **3.046** | **3.050** |
|  | AdoMet(–) | 18 | 0.86 | 0.857 | 0.858 |
|  | AdoHcy(+) | 25 | 1.19 | 1.190 | 1.191 |
|  | AdoHyc(–) | 22 | 1.05 | 1.047 | 1.049 |
|  | Hcy(+) | 24 | 1.14 | 1.142 | 1.144 |
|  | Hcy(–) | 29 | 1.38 | 1.380 | 1.382 |
|  | 5,10-CH_2_-THF(+) | 18 | 0.86 | 0.857 | 0.858 |
|  | 5,10-CH_2_-THF(–) | 22 | 1.05 | 1.047 | 1.049 |
|  | 5-CH_3_-THF(+) | 21 | 1.00 | 0.999 | 1.001 |
|  | 5-CH_3_-THF (–) | 28 | 1.33 | 1.332 | 1.334 |
| Base model/*v*_GNMT_ | Met(+) | 26 | 1.24 | 1.237 | 1.239 |
|  | Met(–) | 22 | 1.05 | 1.047 | 1.049 |
|  | AdoMet(+) | 25 | 1.19 | 1.190 | 1.191 |
|  | AdoMet(–) | 27 | 1.29 | 1.285 | 1.287 |
|  | AdoHcy(+) | 29 | 1.38 | 1.380 | 1.382 |
|  | AdoHyc(–) | 16 | 0.76 | 0.761 | 0.763 |
|  | Hcy(+) | 29 | 1.38 | 1.380 | 1.382 |
|  | Hcy(–) | 21 | 1.00 | 0.999 | 1.001 |
|  | 5,10-CH_2_-THF(+) | 15 | 0.71 | 0.714 | 0.715 |
|  | 5,10-CH_2_-THF(–) | 28 | 1.33 | 1.332 | 1.334 |
| Base model/*v*_CBS_ | Met(+) | 28 | 1.33 | 1.332 | 1.334 |
|  | Met(–) | 26 | 1.24 | 1.237 | 1.239 |
|  | AdoHcy(+) | 28 | 1.33 | 1.332 | 1.334 |
|  | AdoHyc(–) | 18 | 0.86 | 0.857 | 0.858 |
|  | Hcy(+) | 22 | 1.05 | 1.047 | 1.049 |
|  | Hcy(–) | 23 | 1.10 | 1.095 | 1.096 |
|  | 5,10-CH_2_-THF(+) | 24 | 1.14 | 1.142 | 1.144 |
|  | 5,10-CH_2_-THF(–) | 29 | 1.38 | 1.380 | 1.382 |
|  | 5-CH_3_-THF(+) | 22 | 1.05 | 1.047 | 1.049 |
|  | 5-CH_3_-THF (–) | 28 | 1.33 | 1.332 | 1.334 |
| Base model/*v*_MTHFR_ | Met(+) | 19 | 0.90 | 0.904 | 0.906 |
|  | Met(–) | 31 | 1.48 | 1.475 | 1.477 |
|  | Hcy(+) | 28 | 1.33 | 1.332 | 1.334 |
|  | Hcy(–) | 32 | 1.52 | 1.523 | 1.525 |
|  | 5,10-CH_2_-THF(+) | 39 | 1.86 | 1.856 | 1.859 |
|  | 5,10-CH_2_-THF(–) | 21 | 1.00 | 0.999 | 1.001 |
|  | 5-CH_3_-THF(+) | 21 | 1.00 | 0.999 | 1.001 |
|  | 5-CH_3_-THF (–) | 16 | 0.76 | 0.761 | 0.763 |

^a^99%-confidence intervals for Bayes factors were determined by employing jackknife resampling [^48^](#_ENREF_48).

Table S10. Model selection results for the addition of the second interaction

| Model structure | Metabolic effector | Model instances in the posterior | Bayes factor | Lower 99%-conf. value^a^ | Upper 99%-conf. value^a^ |
| --- | --- | --- | --- | --- | --- |
| Base model | ­– | 31 | 1.00 | - | - |
| Base model/*v*_MATIII_ | Met(+) | 39 | 1.26 | 1.257 | 1.259 |
|  | Met(–) | 12 | 0.39 | 0.387 | 0.387 |
|  | AdoHcy(+) | 33 | 1.06 | 1.064 | 1.065 |
|  | AdoHyc(–) | 19 | 0.61 | 0.613 | 0.613 |
|  | Hcy(+) | 21 | 0.68 | 0.677 | 0.678 |
|  | Hcy(–) | 19 | 0.61 | 0.613 | 0.613 |
|  | 5,10-CH_2_-THF(+) | 17 | 0.55 | 0.548 | 0.549 |
|  | 5,10-CH_2_-THF(–) | 20 | 0.65 | 0.645 | 0.646 |
|  | 5-CH_3_-THF(+) | 25 | 0.81 | 0.806 | 0.807 |
|  | 5-CH_3_-THF (–) | 43 | 1.39 | 1.386 | 1.388 |
| Base model/*v*_GNMT_ | Met(+) | 29 | 0.94 | 0.935 | 0.936 |
|  | Met(–) | 23 | 0.74 | 0.742 | 0.743 |
|  | AdoMet(+) | 35 | 1.13 | 1.128 | 1.130 |
|  | AdoMet(–) | 27 | 0.87 | 0.870 | 0.872 |
|  | AdoHcy(+) | 25 | 0.81 | 0.806 | 0.807 |
|  | AdoHyc(–) | 31 | 1.00 | 0.999 | 1.001 |
|  | Hcy(+) | 28 | 0.90 | 0.903 | 0.904 |
|  | Hcy(–) | 29 | 0.94 | 0.935 | 0.936 |
|  | 5,10-CH_2_-THF(+) | 29 | 0.94 | 0.935 | 0.936 |
|  | 5,10-CH_2_-THF(–) | 29 | 0.94 | 0.935 | 0.936 |
| Base model/*v*_CBS_ | Met(+) | 26 | 0.84 | 0.838 | 0.839 |
|  | Met(–) | 27 | 0.87 | 0.870 | 0.872 |
|  | AdoHcy(+) | 19 | 0.61 | 0.613 | 0.613 |
|  | AdoHyc(–) | 26 | 0.84 | 0.838 | 0.839 |
|  | Hcy(+) | 26 | 0.84 | 0.838 | 0.839 |
|  | Hcy(–) | 28 | 0.90 | 0.903 | 0.904 |
|  | 5,10-CH_2_-THF(+) | 21 | 0.68 | 0.677 | 0.678 |
|  | 5,10-CH_2_-THF(–) | 27 | 0.87 | 0.870 | 0.872 |
|  | 5-CH_3_-THF(+) | 25 | 0.81 | 0.806 | 0.807 |
|  | 5-CH_3_-THF (–) | 35 | 1.13 | 1.128 | 1.130 |
| Base model/*v*_MTHFR_ | Met(+) | 22 | 0.71 | 0.709 | 0.710 |
|  | Met(–) | 27 | 0.87 | 0.870 | 0.872 |
|  | Hcy(+) | 37 | 1.19 | 1.193 | 1.194 |
|  | Hcy(–) | 25 | 0.81 | 0.806 | 0.807 |
|  | 5,10-CH_2_-THF(+) | 30 | 0.97 | 0.967 | 0.968 |
|  | 5,10-CH_2_-THF(–) | 23 | 0.74 | 0.742 | 0.743 |
|  | 5-CH_3_-THF(+) | 39 | 1.26 | 1.257 | 1.259 |
|  | 5-CH_3_-THF (–) | 23 | 0.74 | 0.742 | 0.743 |

^a^99%-confidence intervals for Bayes factors were determined by employing jackknife resampling[^48^](#_ENREF_48).

Table S11. Marginal probabilities for similar model structures

| Structures tested | Marginal probability | Lower 99%-conf. value^a^ | Upper 99%-conf. value^a^ |
| --- | --- | --- | --- |
| ∆MTHF(–)/∆AdoHcy(–) | 0.3463 | 0.34630 | 0.34637 |
| ∆MTHF(–)/AadoHcy(–) | 0.3026 | 0.30256 | 0.30260 |
| MTHF(–)/∆AdoHcy(–) | 0.3501 | 0.35010 | 0.35018 |

^a^99%-confidence intervals were determined by jackkniffing expected marginal probabilities over all sampled instances.

Table S12. Bayes factor interpretation adapted from Kass & Raftery[^49^](#_ENREF_49)

| Bayes factor value (*B*_AB_) | Evidence against *m*_B_ and in favour of *m*_A_ |
| --- | --- |
| 1 to 3 | Very week |
| 3 to 20 | Positive |
| 20 to 150 | Strong |
| > 150 | Very strong |

**Supplementary References**

1 Korendyaseva, T. K. *et al.* An allosteric mechanism for switching between parallel tracks in mammalian sulfur metabolism. *Plos Comput Biol* **4**, e1000076, doi:10.1371/journal.pcbi.1000076 (2008).

2 Martinov, M. V., Vitvitsky, V. M., Mosharov, E. V., Banerjee, R. & Ataullakhanov, F. I. A substrate switch: a new mode of regulation in the methionine metabolic pathway. *J Theor Biol* **204**, 521-532, doi:10.1006/jtbi.2000.2035 (2000).

3 Martinov, M. V., Vitvitsky, V. M., Banerjee, R. & Ataullakhanov, F. I. The logic of the hepatic methionine metabolic cycle. *Biochimica et biophysica acta* **1804**, 89-96, doi:10.1016/j.bbapap.2009.10.004 (2010).

4 Nijhout, H. F. *et al.* Long-Range Allosteric Interactions between the Folate and Methionine Cycles Stabilize DNA Methylation Reaction Rate. *Epigenetics* **1**, 81-87 (2006).

5 Prudova, A., Martinov, M. V., Vitvitsky, V. M., Ataullakhanov, F. I. & Banerjee, R. Analysis of pathological defects in methionine metabolism using a simple mathematical model. *Biochimica Et Biophysica Acta-Molecular Basis of Disease* **1741**, 331-338, doi:DOI 10.1016/j.bbadis.2005.04.008 (2005).

6 Reed, M. C., Nijhout, H. F., Sparks, R. & Ulrich, C. M. A mathematical model of the methionine cycle. *J Theor Biol* **226**, 33-43, doi:10.1016/j.jtbi.2003.08.001 (2004).

7 Shlomi, T., Fan, J., Tang, B., Kruger, W. D. & Rabinowitz, J. D. Quantitation of cellular metabolic fluxes of methionine. *Anal Chem* **86**, 1583-1591, doi:10.1021/ac4032093 (2014).

8 Taes, Y. E. C. *et al.* Creatine supplementation decreases homocysteine in an animal model of uremia. *Kidney International* **64**, 1331-1337, doi:DOI 10.1046/j.1523-1755.2003.00206.x (2003).

9 Ozias, M. K. & Schalinske, K. L. All-trans-retinoic acid rapidly induces glycine N-methyltransferase in a dose-dependent manner and reduces circulating methionine and homocysteine levels in rats. *Journal of Nutrition* **133**, 4090-4094 (2003).

10 Horne, D. W. Neither methionine nor nitrous oxide inactivation of methionine synthase affect the concentration of 5,10-methylenetetrahydrofolate in rat liver. *Journal of Nutrition* **133**, 476-478 (2003).

11 Monod, J., Wyman, J. & Changeux, J. P. On the nature of allosteric transitions: a plausible model. *J Mol Biol* **12**, 88-118 (1965).

12 Cabrero, C., Puerta, J. & Alemany, S. Purification and Comparison of 2 Forms of S-Adenosyl-L-Methionine Synthetase from Rat-Liver. *Eur J Biochem* **170**, 299-304, doi:DOI 10.1111/j.1432-1033.1987.tb13699.x (1987).

13 Sullivan, D. M. & Hoffman, J. L. Fractionation and Kinetic-Properties of Rat-Liver and Kidney Methionine Adenosyltransferase Isozymes. *Biochemistry-Us* **22**, 1636-1641, doi:Doi 10.1021/Bi00276a017 (1983).

14 Xu, B., Krudy, G. A. & Rosevear, P. R. Identification of the Metal Ligands and Characterization of a Putative Zinc-Finger in Methionyl-Transfer-Rna Synthetase. *J Biol Chem* **268**, 16259-16264 (1993).

15 Ogawa, H., Gomi, T. & Fujioka, M. Mammalian Glycine N-Methyltransferases - Comparative Kinetic and Structural-Properties of the Enzymes from Human, Rat, Rabbit and Pig Livers. *Comp Biochem Phys B* **106**, 601-611, doi:Doi 10.1016/0305-0491(93)90137-T (1993).

16 Ogawa, H. & Fujioka, M. Purification and Properties of Glycine N-Methyltransferase from Rat-Liver. *J Biol Chem* **257**, 3447-3452 (1982).

17 Yeo, E. J., Briggs, W. T. & Wagner, C. Inhibition of glycine N-methyltransferase by 5-methyltetrahydrofolate pentaglutamate. *J Biol Chem* **274**, 37559-37564, doi:DOI 10.1074/jbc.274.53.37559 (1999).

18 Finkelstein, J. D., Harris, B. J. & Kyle, W. E. Methionine Metabolism in Mammals - Kinetic Study of Betaine-Homocysteine Methyltransferase. *Archives of Biochemistry and Biophysics* **153**, 320-&, doi:Doi 10.1016/0003-9861(72)90451-1 (1972).

19 Jencks, D. A. & Matthews, R. G. Allosteric Inhibition of Methylenetetrahydrofolate Reductase by Adenosylmethionine - Effects of Adenosylmethionine and Nadph on the Equilibrium between Active and Inactive Forms of the Enzyme and on the Kinetics of Approach to Equilibrium. *J Biol Chem* **262**, 2485-2493 (1987).

20 Matthews, R. G. & Daubner, S. C. Modulation of Methylenetetrahydrofolate Reductase-Activity by S-Adenosylmethionine and by Dihydrofolate and Its Polyglutamate Analogs. *Advances in Enzyme Regulation* **20**, 123-131, doi:Doi 10.1016/0065-2571(82)90012-7 (1982).

21 Gorodkin, J. Comparing two K-category assignments by a K-category correlation coefficient. *Comput Biol Chem* **28**, 367-374, doi:10.1016/j.compbiolchem.2004.09.006 (2004).

22 Markham, G. D. & Pajares, M. A. Structure-function relationships in methionine adenosyltransferases. *Cellular and molecular life sciences : CMLS* **66**, 636-648, doi:10.1007/s00018-008-8516-1 (2009).

23 James, F., Nolte, K. D. & Hanson, A. D. Purification and Properties of S-Adenosyl-L-Methionine-L-Methionine S-Methyltransferase from Wollastonia-Biflora Leaves. *J Biol Chem* **270**, 22344-22350 (1995).

24 Huang, Y. *et al.* Mechanisms for auto-inhibition and forced product release in glycine N-methyltransferase: Crystal structures of wild-type, mutant R175K and S-adenosylhomocysteine-bound R175K enzymes. *J Mol Biol* **298**, 149-162, doi:DOI 10.1006/jmbi.2000.3637 (2000).

25 Takata, Y. *et al.* Catalytic mechanism of glycine N-methyltransferase. *Biochemistry-Us* **42**, 8394-8402, doi:Doi 10.1021/Bi034245a (2003).

26 Tehlivets, O., Malanovic, N., Visram, M., Paykov-Keller, T. & Keller, W. S-adenosyl-L-homocysteine hydrolase and methylation disorders: Yeast as a model system. *Biochimica Et Biophysica Acta-Molecular Basis of Disease* **1832**, 204-215, doi:DOI 10.1016/j.bbadis.2012.09.007 (2013).

27 Chen, Z., Crippen, K., Gulati, S. & Banerjee, R. Purification and kinetic mechanism of a mammalian methionine synthase from pig liver. *The Journal of biological chemistry* **269**, 27193-27197 (1994).

28 Borcsok, E. & Abeles, R. H. Mechanism of Action of Cystathionine Synthase. *Archives of Biochemistry and Biophysics* **213**, 695-707, doi:Doi 10.1016/0003-9861(82)90600-2 (1982).

29 Trimmer, E. E., Ballou, D. P. & Matthews, R. G. Methylenetetrahydrofolate reductase from Escherichia coli: elucidation of the kinetic mechanism by steady-state and rapid-reaction studies. *Biochemistry-Us* **40**, 6205-6215 (2001).

30 Vanoni, M. A. & Matthews, R. G. Kinetic Isotope Effects on the Oxidation of Reduced Nicotinamide Adenine-Dinucleotide Phosphate by the Flavoprotein Methylenetetrahydrofolate Reductase. *Biochemistry-Us* **23**, 5272-5279, doi:Doi 10.1021/Bi00317a027 (1984).

31 Flamholz, A., Noor, E., Bar-Even, A. & Milo, R. eQuilibrator-the biochemical thermodynamics calculator. *Nucleic Acids Res* **40**, 770-775, doi:10.1093/nar/gkr874 (2012).

32 Finkelstein, J. D., Kyle, W. E., Harris, B. J. & Martin, J. J. Methionine Metabolism in Mammals - Concentration of Metabolites in Rat-Tissues. *Journal of Nutrition* **112**, 1011-1018 (1982).

33 Finkelstein, J. D. & Martin, J. J. Methionine Metabolism in Mammals - Adaptation to Methionine Excess. *J Biol Chem* **261**, 1582-1587 (1986).

34 Jacobs, R. L., Stead, L. M., Brosnan, M. E. & Brosnan, J. T. Hyperglucagonemia in rats results in decreased plasma homocysteine and increased flux through the transsulfuration pathway in liver. *J Biol Chem* **276**, 43740-43747, doi:DOI 10.1074/jbc.M107553200 (2001).

35 Chen, L. X. *et al.* Impaired liver regeneration in mice lacking methionine adenosyltransferase 1A. *Faseb J* **18**, 914-+, doi:DOI 10.1096/fj.03-1204fje (2004).

36 Wolfe, J. in *eLS* (John Wiley & Sons, Ltd, 2001).

37 Kukko, E. & Heinonen, J. The intracellular concentration of pyrophosphate in the batch culture of Escherichia coli. *European journal of biochemistry / FEBS* **127**, 347-349 (1982).

38 Bevington, A. *et al.* A Study of Intracellular Ortho-Phosphate Concentration in Human-Muscle and Erythrocytes by P-31 Nuclear-Magnetic-Resonance Spectroscopy and Selective Chemical-Assay. *Clinical Science* **71**, 729-735 (1986).

39 Regina, M., Korhonen, V. P., Smith, T. K., Alakuijala, L. & Eloranta, T. O. Methionine Toxicity in the Rat in Relation to Hepatic Accumulation of S-Adenosylmethionine - Prevention by Dietary Stimulation of the Hepatic Transsulfuration Pathway. *Archives of Biochemistry and Biophysics* **300**, 598-607, doi:DOI 10.1006/abbi.1993.1083 (1993).

40 Scheer, J. B., Mackey, A. D. & Gregory, J. F. Activities of hepatic cytosolic and mitochondrial forms of serine hydroxymethyltransferase and hepatic glycine concentration are affected by vitamin B-6 intake in rats. *Journal of Nutrition* **135**, 233-238 (2005).

41 Vitvitsky, V. *et al.* Testosterone regulation of renal cystathionine beta-synthase: implications for sex-dependent differences in plasma homocysteine levels. *American Journal of Physiology-Renal Physiology* **293**, F594-F600, doi:DOI 10.1152/ajprenal.00171.2007 (2007).

42 Bontemps, F., Vincent, M. F. & Vandenberghe, G. Mechanisms of Elevation of Adenosine Levels in Anoxic Hepatocytes. *Biochem J* **290**, 671-677 (1993).

43 Desanchez, V. C. *et al.* 24-Hour Changes of S-Adenosylmethionine, S-Adenosylhomocysteine Adenosine and Their Metabolizing Enzymes in Rat-Liver - Possible Physiological Significance in Phospholipid Methylation. *International Journal of Biochemistry* **23**, 1439-1443, doi:Doi 10.1016/0020-711x(91)90287-W (1991).

44 Garrow, T. A. Purification, kinetic properties, and cDNA cloning of mammalian betaine-homocysteine methyltransferase. *J Biol Chem* **271**, 22831-22838 (1996).

45 Millian, N. S. & Garrow, T. A. Human betaine-homocysteine methyltransferase is a zinc metalloenzyme. *Archives of Biochemistry and Biophysics* **356**, 93-98, doi:DOI 10.1006/abbi.1998.0757 (1998).

46 Singh, S., Padovani, D., Leslie, R. A., Chiku, T. & Banerjee, R. Relative Contributions of Cystathionine beta-Synthase and gamma-Cystathionase to H2S Biogenesis via Alternative Trans-sulfuration Reactions. *J Biol Chem* **284**, 22457-22466, doi:DOI 10.1074/jbc.M109.010868 (2009).

47 Veech, R. L., Egglesto.Lv & Krebs, H. A. Redox State of Free Nicotinamide-Adenine Dinucleotide Phosphate in Cytoplasm of Rat Liver. *Biochem J* **115**, 609-& (1969).

48 Miller, R. G. Jackknife - Review. *Biometrika* **61**, 1-15 (1974).

49 Kass, R. E. & Raftery, A. E. Bayes Factors. *J Am Stat Assoc* **90**, 773-795, doi:Doi 10.1080/01621459.1995.10476572 (1995).
